# The structural mechanism of dimeric DONSON in replicative helicase activation

**DOI:** 10.1016/j.molcel.2023.09.029

**Published:** 2023-10-10

**Authors:** Milos A. Cvetkovic, Paolo Passaretti, Agata Butryn, Alicja Reynolds-Winczura, Georgia Kingsley, Aggeliki Skagia, Cyntia Fernandez-Cuesta, Divyasree Poovathumkadavil, Roger George, Anoop S. Chauhan, Satpal S. Jhujh, Grant S. Stewart, Agnieszka Gambus, Alessandro Costa

**Affiliations:** 1 Macromolecular Machines Laboratory, The Francis Crick Institute, London NW1 1AT, UK; 2 Institute of Cancer and Genomic Sciences, Birmingham Centre for Genome Biology, University of Birmingham, Birmingham B15 2TT, UK; 3 Structural Biology Science Technology Platform, The Francis Crick Institute, London NW1 1AT, UK

## Abstract

The MCM motor of the replicative helicase is loaded onto origin DNA as an inactive double hexamer before replication initiation. Recruitment of activators GINS and Cdc45 upon S-phase transition promotes the assembly of two active CMG helicases. Although work with yeast established the mechanism for origin activation, how CMG is formed in higher eukaryotes is poorly understood. Metazoan Downstream neighbor of Son (DONSON) has recently been shown to deliver GINS to MCM during CMG assembly. What impact this has on the MCM double hexamer is unknown. Here, we used cryoelectron microscopy (cryo-EM) on proteins isolated from replicating *Xenopus* egg extracts to identify a double CMG complex bridged by a DONSON dimer. We find that tethering elements mediating complex formation are essential for replication. DONSON reconfigures the MCM motors in the double CMG, and primordial dwarfism patients’ mutations disrupting DONSON dimerization affect GINS and MCM engagement in human cells and DNA synthesis in *Xenopus* egg extracts.

## Graphical abstract

**Graphical abstract:**
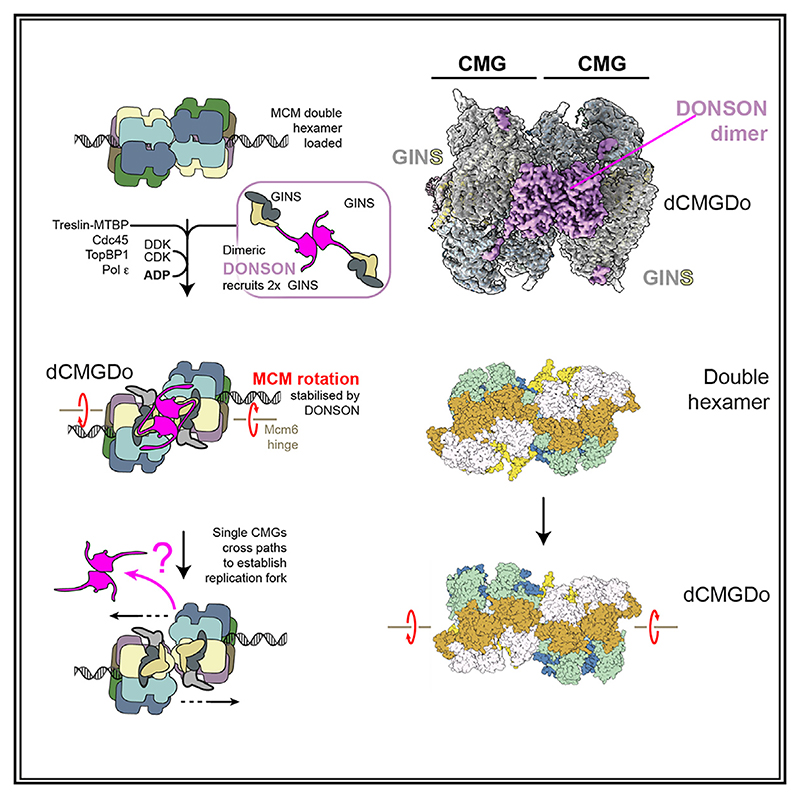

## Introduction

DNA replication is essential for cell proliferation. It occurs only once per cell cycle, and errors in the control mechanisms promote chromosomal instability, causing genetic disease and cancer. To ensure that DNA is copied only once, eukaryotic cells temporally separate DNA loading of the minichromosome maintenance (MCM) replicative helicase from its activation.^1,2^ MCM is a ring-shaped ATPase that becomes loaded as a double hexamer onto origin DNA before replication initiation.^3–7^ The DNA unwinding function in the double hexamer is inactive. Activation requires the recruitment of a set of firing factors, including GINS and Cdc45 that bind MCM to assemble the functional form of the helicase, named CMG (Cdc45-MCM-GINS).^8–11^ Genetics and biochemical reconstitution with purified proteins provided a clear picture of the pathway and molecular mechanism for CMG formation and activation in yeast.^2,12^ This work established that CMG assembly can be staged in three steps, namely double-hexamer phosphorylation, Cdc45 recruitment, and GINS recruitment. In the first step, double hexamers are phosphorylated by the Dbf4-dependent kinase (DDK).^13–19^ The second step toward CMG assembly is Cdc45 recruitment, mediated by Sld3 (bound to Sld7), which specifically recognizes double hexamer phosphorylation written by DDK.^20,21^ GINS recruitment instead depends on the activity of cyclin-dependent kinase Clb5-Cdc28 (CDK) that phosphorylates Sld2, promoting its association with Dpb11, Pol epsilon, and GINS. CDK also phosphorylates Sld3, and this modification is also read by Dpb11. Concomitant recognition of phospho-Sld2 and phospho-Sld3 by Dpb11 recruits GINS to the double hexamer.^22–24^ Cdc45 and GINS engagement promotes ATP binding by MCM, which in turn triggers release of Dpb11, Sld3/7, and Sld2.^25^

Structural work established that GINS and Cdc45 are recruited to form two CMG complexes in a step known to require the Pol epsilon replicative polymerase.^25,26^ This results in a double CMG (dCMG)-Pol epsilon assembly that distorts the DNA double helix and changes in the register of dimerizing MCM rings. As the two rings rotate with respect to one another, the two N-terminal domains of MCM become almost fully detached, remaining tethered via the Mcm6 zinc finger (ZnF) domain and exposing B-form duplex DNA between the two hexamers. Within each helicase ring, ATP binding triggers a change in the DNA grip, leading to untwisting and melting of the double helix.^27^ In a subsequent step, Mcm10 activates the ATP hydrolysis function of MCM, causing the two helicases to eject one DNA strand from their central channel and cross paths, so that the strand ejected from one hexamer becomes the translocation strand of the other.^25,28,29^ This mechanism requires that both helicases are activated at the same time for bidirectional replication to occur. Structural evidence supports this symmetric helicase activation mechanism. In fact, cryoelectron microscopy (cryo-EM) reconstructions obtained from biochemical reconstitution efforts, or isolation of chromatin-bound complexes from cells, show that dimeric MCM assemblies can either be found as symmetric inactive double hexamers or dCMG-Pol epsilon complexes that nucleate DNA melting symmetrically.^6,7,27,30,31^ Never was an asymmetric intermediate observed, which contains an inactive MCM bound to an activated CMG-Pol epsilon. Although key for the establishment of bidirectional replication, the structural mechanism for the conversion from double hexamer to dCMG remains only partially understood. Furthermore, only for yeast but not for higher eukaryotes has the complete list of factors required for CMG assembly and activation been determined. In fact, work with higher eukaryotes led to identifying almost all orthologs of the factors involved in yeast replication initiation, including the Dpb11 homolog, TopBP1, and the Sld3-7 homolog, Treslin-MTBP.^32–34^ The exception is a homolog for yeast Sld2, whose identity remains unclear. Previous studies noted that the RecQL4 factor, which contains an Sld2 homology domain, is essential for replication initiation. However, RecQL4 is not required for Cdc45 and GINS interaction with MCMs, rather it acts downstream of CMG formation.^35–40^ Recent work with frog egg extracts led to the suggestion that DONSON (Downstream neighbor of Son) is the elusive functional homolog of yeast Sld2 in higher eukaryotes. Like Sld2, DONSON directly contacts GINS, and this interaction supports CMG assembly and triggers DNA replication initiation. Moreover, like Sld2, chromatin association by DONSON requires double hexamer formation, the kinase activity of DDK and CDK as well as the presence of TopBP1/Dpb11.^41,42^ Mutations in DONSON are the cause of microcephalic primordial dwarfism, a group of disorders characterized by growth delays, often related to defects in the initiation step of DNA replication.^43,44^ A role for DONSON in origin activation is remarkable as it indicates a function separate from its established role during elongation. In fact, DONSON was previously implicated in processes required after origin activation, including proper activation of S-phase checkpoint response, replication fork protection from the action of nucleases, and traversal of inter-strand cross-links.^43,45^ To understand the role of DONSON in GINS recruitment and the symmetric activation of the MCM replicative helicase, we used the *Xenopus laevis* egg extract system to stall replication at an early stage and imaged replication protein assemblies isolated from chromatin with cryo-EM.

## Results

### CMG assemblies isolated from replicating frog egg extracts

**Figure 1:**
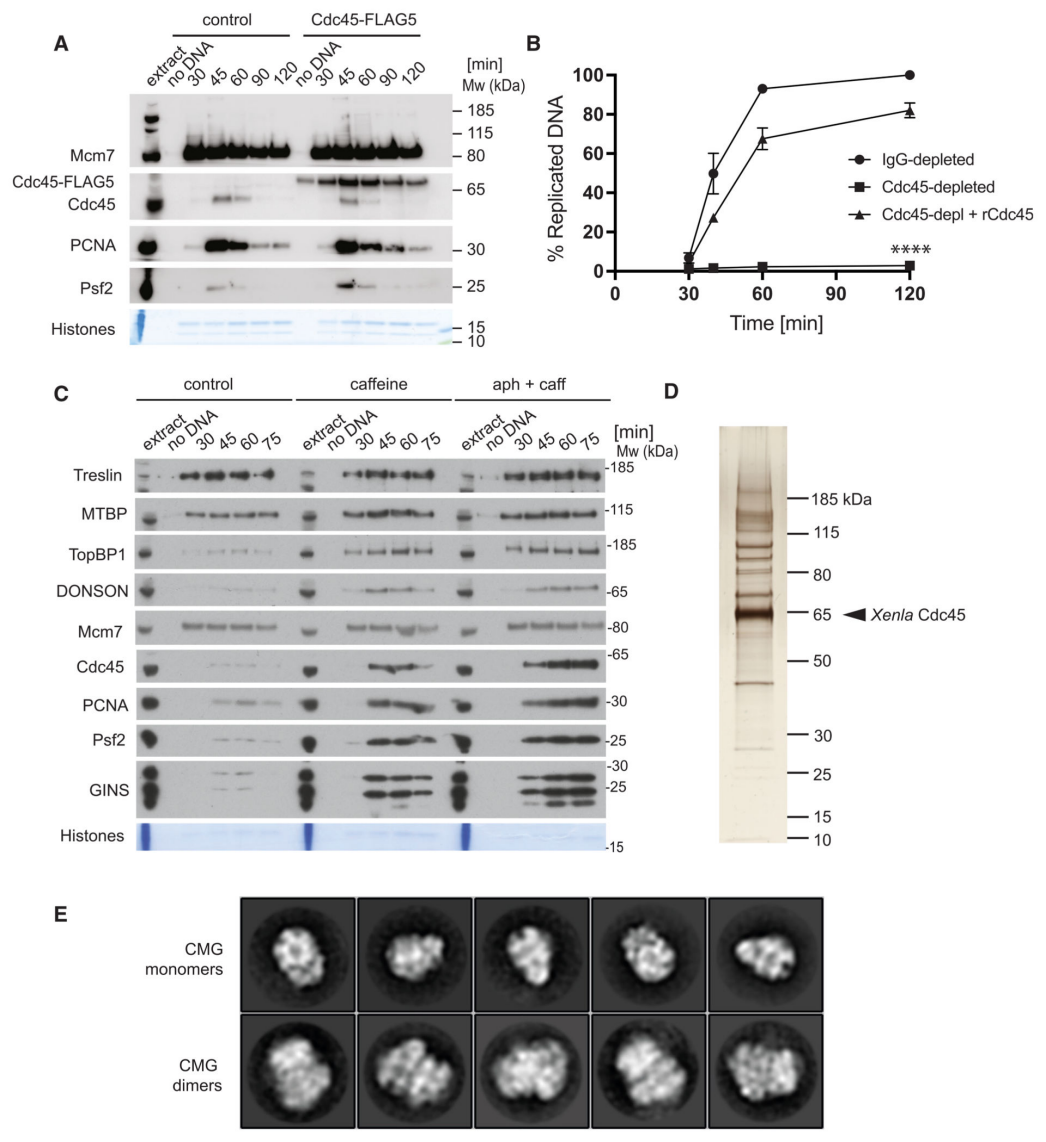
Isolation of replication complexes from chromatin replicated in *Xenopus laevis* egg extract (A). Cdc45-TEV-His_10_-FLAG_5_ becomes chromatin incorporated. DNA replication reaction was set up in *Xenopus* egg extract with optional addition of 70 nM Cdc45-TEV-His_10_-FLAG_5_, and chromatin was isolated at indicated times. Chromatin-bound factors were resolved on SDS-PAGE and immunoblotted with indicated antibodies. ‘No DNA’ control served as chromatin specificity control, whereas Coomassie stained histones serve as a loading and purity control. (B). DNA replication reaction was set up in IgG-or Cdc45-depleted extract optionally supplemented with recombinant Cdc45-TEV-His_10_-FLAG_5_ as in (A). The synthesis of nascent DNA was followed by incorporation of α^32^P-dATP into newly synthesized DNA. n = 3, mean with SEM presented. Two-way ANOVA comparing to IgG-depleted, **** p < 0.0001. (C). DNA replication reaction was set up in *Xenopus* egg extract with optional addition of 5 mM caffeine and 40 µM aphidicolin. Chromatin was isolated at indicated times and analyzed as in (A). (D). DNA replication reaction was set up in *Xenopus* egg extract with optional addition of 70 nM Cdc45-TEV-His_10_-FLAG_5_, 5 mM caffeine and 40 µM aphidicolin. After 60 min of the reaction, chromatin was isolated, digested with benzonase, and Cdc45-TEV-His_10_-FLAG_5_ immunoprecipitated with M2 FLAG beads. The immunoprecipitated sample was resolved on SDS-PAGE and silver stained. (E). Negative stain 2D averages of single (first row) and double CMG (second row).

To understand the mechanism for helicase activation that supports replication origin firing in vertebrates, we devised a protocol to isolate the activated replicative helicase from replication reactions established using the *Xenopus laevis* egg extract system. First, we used bacterial overexpression to produce *Xenla*Cdc45 fused to C-terminal, tobacco etch virus (TEV) protease-cleavable, tandem His_10_-FLAG_5_ tags (Figure S1). Demembranated sperm DNA was added to an activated egg extract supplemented with recombinant Cdc45. Cdc45-TEV-His_10_-FLAG_5_ becomes incorporated into isolated chromatin (Figure 1A) and rescues DNA replication in extracts depleted for the endogenous protein (Figure 1B). We repeated the replication experiments in the presence of caffeine-and-aphidicolin to capture an early step in replication. Caffeine shuts down the DNA damage checkpoint by inhibiting the two master control kinases in DNA damage response, ataxia telangiectasia mutated (ATM) and ataxia telangiectasia and Rad3-Related (ATR).^46^ Aphidicolin is a potent inhibitor of family B DNA polymerases (including the Pol alpha, delta, and epsilon replicative polymerases).^47^ The combined use of these two inhibitors leads to uncontrolled origin firing but stops DNA synthesis from happening. In these conditions, we expected accumulation of stable intermediates of replication initiation and stalled replication forks. In fact, western blot analysis performed on isolated chromatin showed a marked increase in initiation factors and components of the established replisome when caffeine or caffeine/aphidicolin were used (Figure 1C). After supplementing these egg extracts with recombinant Cdc45, we isolated chromatin, digested DNA using benzonase, and immunoprecipitated Cdc45, using anti-FLAG M2 beads. Silver-stain SDS-PAGE revealed multiple proteins co-purified with recombinant Cdc45 (Figure 1D), and mass spectrometry analysis identified several replication factors (Table S1). These include all six subunits of the MCM complex (within the first ten hits), the four GINS subunits, RPA, and components of the replicative polymerase assemblies, Pol alpha/primase, Pol epsilon, and Pol delta. Hence, recombinant Cdc45 can be incorporated in a CMG complex competent for replication fork establishment. Mass spectrometry also identified DONSON as the member of a Cdc45-containing assembly. To establish whether the Cdc45-containing complexes represented structural intermediates of origin activation or rather the established replication fork, we performed negative stain electron microscopy combined with single-particle two-dimensional (2D) analysis. The resulting 2D averages revealed recognizable views of a single CMG, similar to the negative stain averages observed after origin firing reconstituted *in vitro* with purified yeast proteins.^25^ We also observed two CMGs with N-terminal MCM sides engaged in dimerization, which were highly reminiscent of the initiation intermediate, dCMG-Pol epsilon, caught in the act of nucleating DNA melting before helicases cross paths^27^ (Figure 1E). From our negative stain analysis, it was unclear whether either of the two CMG assemblies was found associated with DONSON or any components of the established replication fork.

### A dimeric CMG-DONSON complex revealed by cryo-EM

Seeking to characterize the dimeric CMG assemblies, we repeated the same electron microscopy experiment in cryogenic conditions. 2D classification combined with *ab initio* structure determination and three-dimensional (3D) classification confirmed the presence of single CMG and dCMG assemblies (Figure S2).

**Figure 2:**
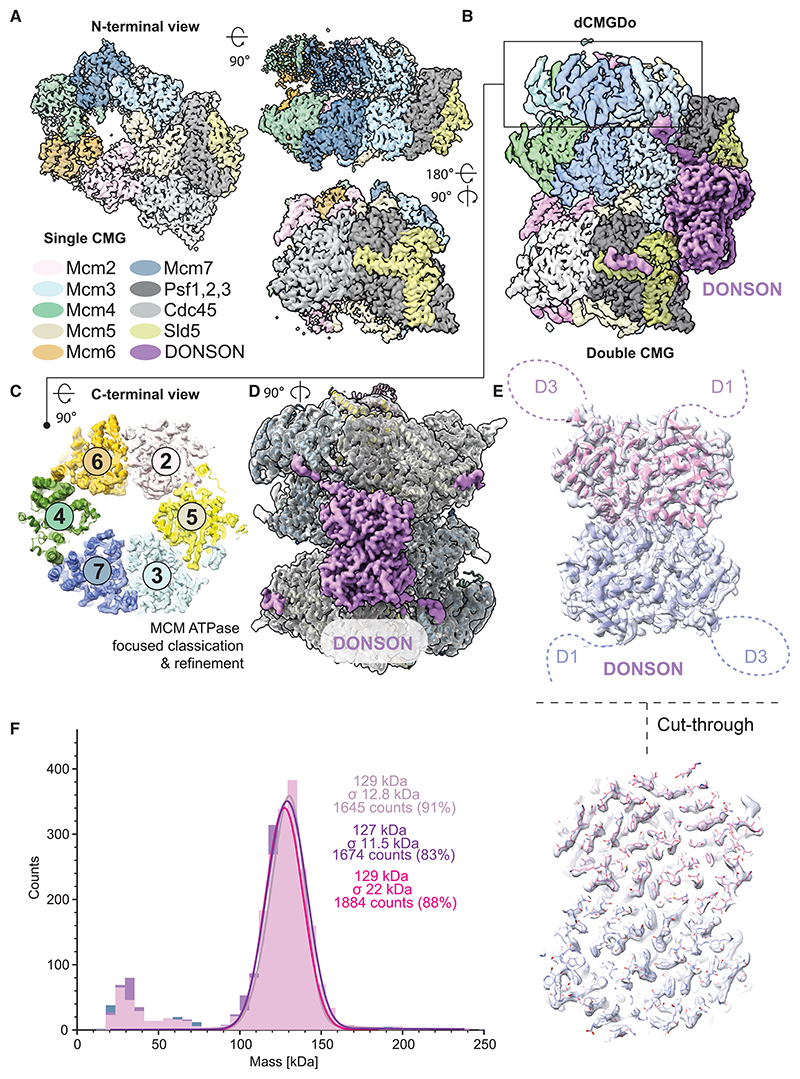
Cryo-EM analysis of Cdc45 containing complexes leads to identification of a DONSON-bound double CMG assembly (A). The N-terminal end-on view, the side, and the rotated side view of single CMG. (B). The side view of a double CMG in a configuration distinct from what was previously observed with the yeast CMG assembled at an origin of replication. (C) A view of the MCM ATPase improved after focused classification and refinement. (D) Unassigned density after building the double CMG atomic model. (E) Cartoon representation of a DONSON homodimer built into the cryo-EM density represented with transparent surface rendering and a cut-through view. (F) Mass photometry analysis demonstrates that purified recombinant DONSON homodimerizes.

The structure of the single CMG was refined to 3.0 Å average resolution, with 2.6 Å local resolution for the GINS, Cdc45, and part of N-terminal MCM, whereas the ATPase domain was more flexible (Figures 2A and S3). No DNA density was observed bound to CMG, as expected given that Cdc45-containing complexes were purified from chromatin after DNA digestion using benzonase. Neither DONSON nor any of the other replication fork components were observed, possibly because their association with CMG involves flexible elements or because these factors had become dissociated in the conditions used during purification and cryo-EM sample preparation.

The structure of dCMG contained 2-fold symmetry across the dimerizing N-terminal domain and was refined to 3.1 Å average resolution (Figure 2B). As for the single CMG, the highest local resolution (2.7–3.7 Å) was detected for the N-terminal MCM region, GINS and Cdc45, whereas the ATPase domain was more flexible (Figure S3). Resolution of the ATPase tier was improved to 3.5 Å after performing symmetry expansion, signal subtraction, and focused classification on one ATPase domain, and refinement of the most populated conformers (Figures 2C, S2, and S3). Although no DNA was found in the complex, an extensive globular density feature, resolved to 2.7 Å, could be recognized straddling across two CMG assemblies (Figure 2D). Within each CMG assembly, this density bridges between Mcm3 and GINS. Because of the GINS interaction, we speculated that the bridging element could correspond to the DONSON factor that promotes incorporation of GINS into the CMG complex. To test our hypothesis, we docked the AlphaFold2 model for a DONSON monomer into the cryo-EM density left unoccupied in the dCMG complex.^48^ Automated docking revealed two non-overlapping solutions, with identical cross-correlation scores, showing that the unoccupied density in the dCMG complex corresponds to a 2-fold symmetric homodimer of DONSON (Figure 2E). We named this 24-mer complex dCMGDo (for double CMG-DONSON) and subjected the complete atomic structure to real space refinement (Table 1).

### A constitutive DONSON homodimer engages two GINS sites

Dimeric DONSON could deposit two GINS assemblies onto a phosphorylated double hexamer on the path to CMG formation. Alternatively, monomeric DONSON could deliver two GINS assemblies in independent events and homo-dimerize only when incorporated in the dCMGDo. To discriminate between these two possibilities, we overexpressed and purified recombinant *Xenopus laevis* DONSON from *E. coli* cells. Mass photometry analysis showed that recombinant DONSON is primarily dimeric, having a measured molecular mass of 128.3 ±15.4 kDa, in striking agreement with what is expected for a DONSON homodimer (132 kDa, Figure 2F). A truncation variant that lacks residues 1–155, forming the unstructured D1 loop, still assembles as a homodimer (Figure S4A). Thus, isolated recombinant DONSON homodimerizes via the globular domain, as seen in the dCMGDo assembly.

**Figure 3:**
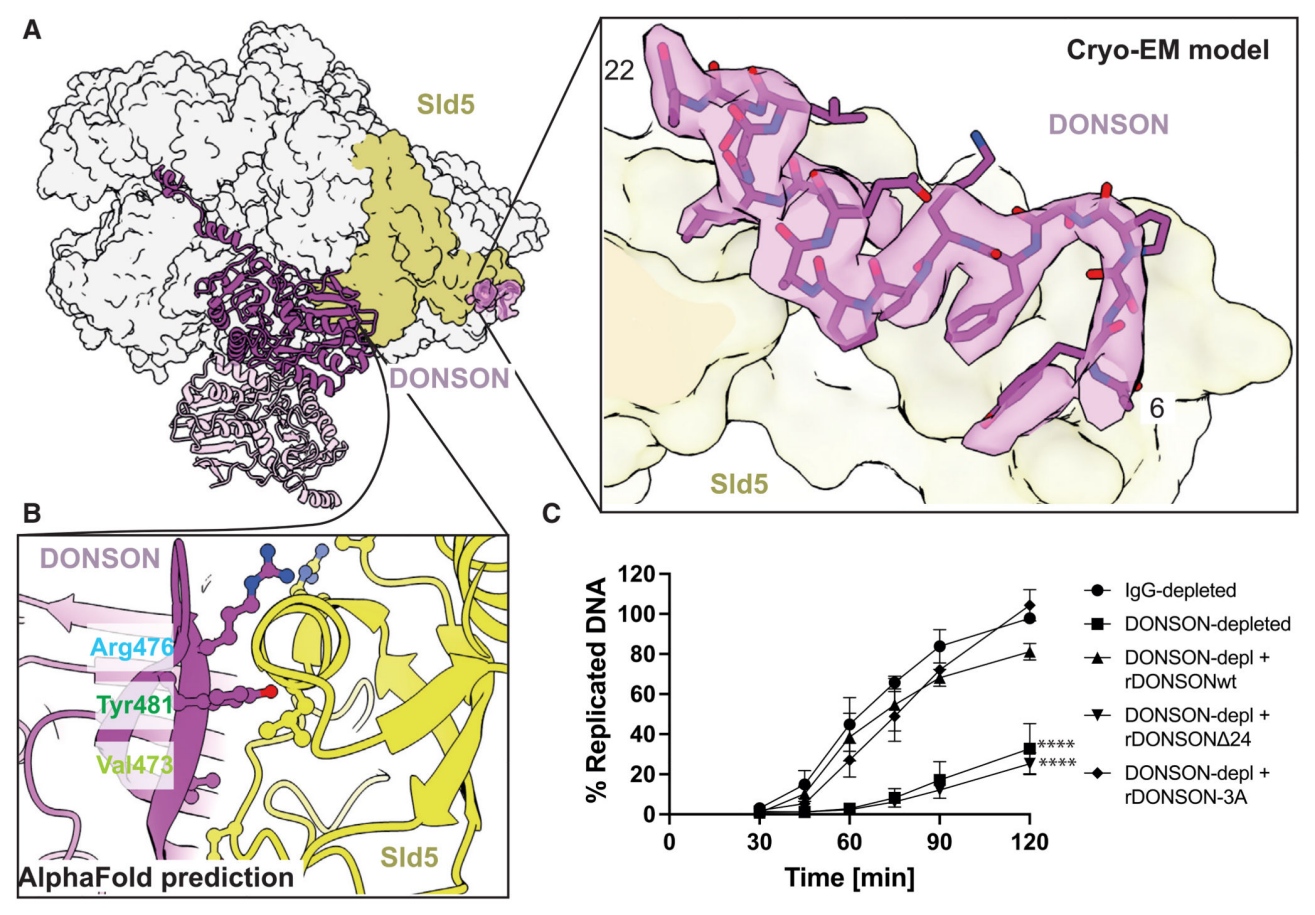
DONSON binding to Sld5 (A) One isolated CMG from the dCMGDo structure, engaged by dimeric DONSON. Only one DONSON protomer binds one CMG. The zoomed in view shows the N-terminal tail of DONSON, tethered to the DONSON core via an unstructured (invisible) linker domain, and bound to Sld5. (B) AlphaFold prediction of the engagement interface between the DONSON globular dimerization domain and the Sld5 B-domain. (C) DNA replication reaction was set up in IgG-or DONSON-depleted extract optionally supplemented with 16 nM recombinant wild-type or indicated DONSON mutants. The synthesis of nascent DNA was followed by incorporation of α ^32^P-dATP into newly synthesized DNA at indicated times. n = 3, mean with SEM presented. Two-way ANOVA comparing with IgG-depleted, **** p < 0.0001.

To understand the interaction between GINS and DONSON, we analyzed the cryo-EM structure in detail. We found that DONSON contacts two separate sites on GINS. A first interface involves the A-domain of Sld5, which we found decorated by unoccupied density. AlphaFold2 multimer predicted a high-confidence interaction between this Sld5 domain and residues 7–24 of DONSON.^48^ Based on the cryo-EM density, we could build an accurate atomic model for residues 6–22, which strikingly matches the predicted structure (Figure 3A). The second interface is provided by the B-domain of Sld5, engaged by a hairpin loop (amino acids 473–482), emanating from the DONSON central core (Figure 3B). Single residues involved in this interface cannot be clearly identified based on the cryo-EM map. In fact, the contact between the two surfaces could only be observed in the non-sharpened density map. Nonetheless, AlphaFold2 multimer predicted this interface with high confidence, providing a model that accurately recapitulates the relative orientation between GINS and DONSON, observed by cryo-EM.^48^ To assess the importance of these interactions, we generated two DONSON variants, with changes in elements interacting with Sld5 A-domain and B-domain, respectively. We named these variants DONSONΔ24 (containing a truncation of residues 1–24) and DONSON 3A (containing changes Val473Ala, Arg476Ala, and Tyr481Ala) (Figure S1B). To ensure that these changes do not alter DONSON’s core structure, we analyzed both variants using mass photometry. We found that both DONSONΔ24 as well as DONSON 3A form homodimers, like the wild-type (WT) protein (Figures S4B and S4C). Thus, any defect in DONSON’s function should be ascribed to defective GINS interaction and not protein misfolding. To test the functionality of our mutants during DNA replication, we immunodepleted DONSON from the egg extract using an antibody known to deplete endogenous DONSON to levels below 5%, which strongly diminishes the extract’s ability to synthesize nascent DNA.^42^ We found that although WT recombinant DONSON purified from *E. coli* rescued replication to the levels of control IgG-depleted extract, DONSONΔ24 was unable to support DNA replication in the DONSON-depleted extract. However, DONSON 3A variant was proficient in supporting nascent DNA synthesis (Figure 3C), in agreement with the cryo-EM observation that the hairpin loop of DONSON is flexible and only loosely engages the Sld5 B-domain. We confirmed that WT DONSON and DONSON 3A supported loading of Cdc45 and GINS onto chromatin, but DONSONΔ24 was defective (Figure S5A). To test the prediction that a DONSON variant lacking the N-terminal Sld5 interaction element fails to engage GINS, we immunoprecipitated recombinant FLAG-tagged GINS after incubation with different DONSON variants and probed the interaction with an anti-DONSON antibody. We found that WT DONSON and DONSON 3A could associate with recombinant GINS, but DONSONΔ24 failed to interact (Figure S5B).

### Dimeric DONSON engages two Mcm3 sites

**Figure 4:**
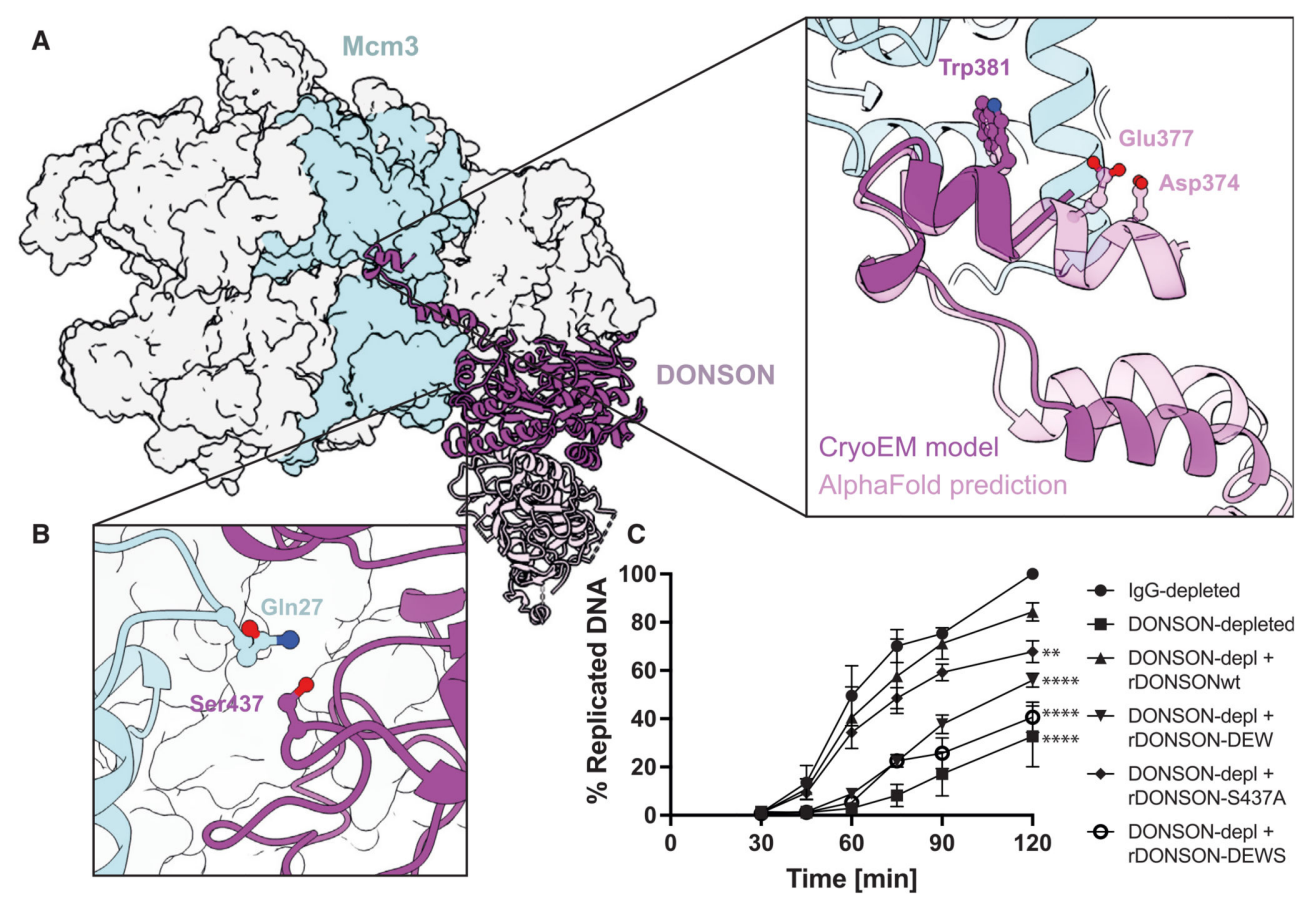
DONSON binding to Mcm3 (A) One isolated CMG from the dCMGDo structure, engaged by dimeric DONSON. The zoomed in view shows the D3 loop of DONSON engaging the Mcm3 ATPase domain. The solid cartoon represents the atomic model built into the cryo-EM density. The transparent cartoon represents the AlphaFold prediction. The two models identify Trp381 as an Mcm3 ATPase interacting element. AlphaFold also identifies Asp374 and Glu 377 as ATPase-engaged. (B) Ser437 engages Mcm3 A-domain residue, Gln27, according to the atomic model built into the cryo-EM density. (C) DNA replication reaction was set up in IgG-or DONSON-depleted extract optionally supplemented with 16 nM recombinant wild-type or indicated DONSON mutants. The synthesis of nascent DNA was followed by incorporation of a^32^P-dATP into newly synthesized DNA at indicated times. n = 3, mean with SEM presented. Two-way ANOVA comparing to IgG-depleted, **** p < 0.0001. ** p = 0.0046.

We then analyzed the interaction between MCM and DONSON in the dCMGDo cryo-EM structure. A first interaction involves the outer perimeter of the ATPase domain of Mcm3 and an alpha helix element inserted within a flexible loop (D3 loop, residues 345–395) emanating from the DONSON core. This DONSON alpha helix features a well-resolved Trp381 engaging Mcm3 Lys285. Our model strikingly resembles the AlphaFold2 multimer model,^48^ which also predicted additional Mcm3 interactions with DONSON residues Asp374 and Glu377 (not well resolved in the experimental map, Figure 4A). A second interaction involves the N-terminal A-domain of Mcm3, at a site proximal to that engaged by GINS subunit Psf3 in the CMG (Figure 4B). To test the importance of the interaction sites with Mcm3, we generated three DONSON variants. The DONSON DEW variant, containing Asp374, Glu377, and Trp381 changed to alanine, targeted the Mcm3 ATPase domain interaction (Figure 4A). Ser437Ala targeted a polar interaction of DONSON with Mcm3 Gln27, embedded in a pocket that appears otherwise hydrophobic (Figure 4B). A third variant, DONSON DEWS, combined DONSON DEW and Ser437Ala. We confirmed that these three variants can homodimerize like the WT proteins (Figures S4D–S4F) and then asked whether changes in the DONSON-Mcm3 interaction had any effect on DNA replication. Recombinant DONSON containing the Ser437Ala mutation showed a mild defect in rescuing DNA replication after immuno-depletion of the endogenous protein. Instead, the DONSON DEW variant was severely, although not totally, deficient at rescuing replication. The DONSON DEWS variant, which targeted both N-terminal and ATPase interaction sites on Mcm3, was completely unable to rescue replication (Figure 4C). We confirmed that DONSON Ser437Ala supported loading of Cdc45 and GINS onto chromatin, but DONSON DEW and DONSON DEWS were defective (Figure S5C).

### MCM dimerization in dCMGDo

**Figure 5:**
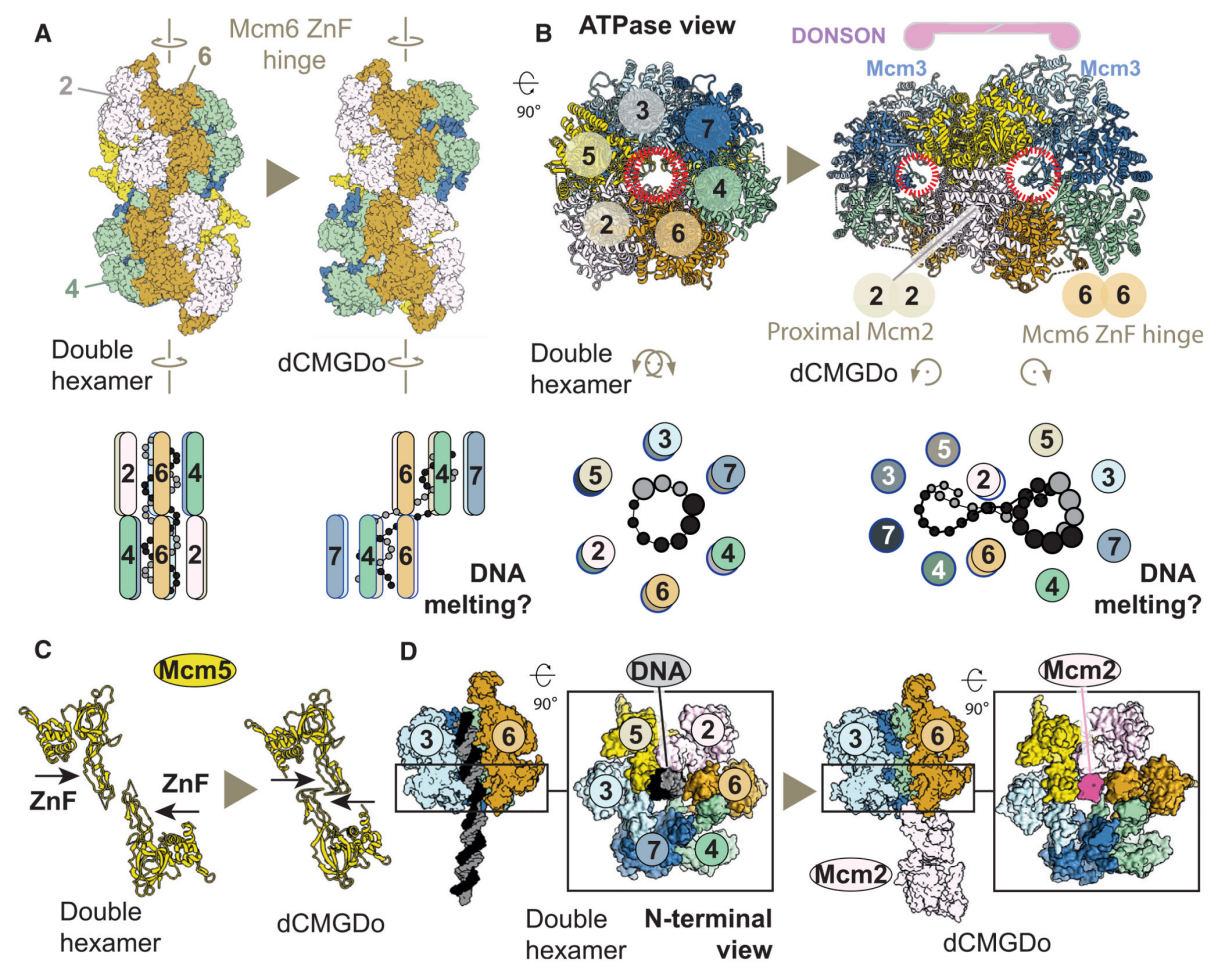
Conformational changes within the MCM occurring upon conversion from double hexamer to dCMGDo (A) Two MCM rings, viewed from the side, rotate clockwise with respect to one another pivoting around Mcm6. This movement causes the Mcm7 side of the ring to swing out. This movement agrees with a theoretical model proposed previously.^49^ (B) The double hexamer to dCMGDo conversion viewed from an end-on view of the ATPase ring. The two MCM central channels are no longer co-axial in dCMGDo. (C) Two N-terminal ZnF domains cross paths upon conversion from double hexamer to dCMGDo. (D) Reconfiguration of the N-terminal MCM ring pore upon double hexamer to dCMGDo transition. Mcm2 from one ring in dCMGDo occludes the MCM central channel occupied by duplex DNA in the double hexamer.

We compared the MCM dimerization interface in dCMGDo with dimeric complexes observed before^6,7,17,27,30,31^ and found that the hexamer interface is completely reconfigured upon DONSON binding. First, the inter-ring interactions are significantly loosened. In fact, by measuring the distance between the N-terminal ZnF domains, we conclude that the two MCM rings hover over one another rather than engaging in direct interactions. With the shortest inter-ring distances ranging from 5 to 7 Å, any contacts, if they exist, are likely water mediated. We then inspected the complex from the C-terminal ATPase side to observe the structural changes upon double hexamer to dCMGDo conversion. We detected an ~60° clockwise rotation of one MCM ring pivoting around two aligned N-terminal ZnF domains of Mcm2 and Mcm6 across the double hexamer (Figure 5A; Video S1). As a result, the Mcm7 side of one hexamer swings out causing the two MCM rings to become misaligned, to the point that the two central channels are no longer co-axial and rather identify topologically separate entities (Figure 5B). This reconfiguration moves the two Mcm5 ZnF domains past one other, whereas they still remain in close proximity (Figure 5C). The ZnF domains of two Mcm2 subunits move from a distal to a proximal position at the inter-ring interface. In this configuration, the Mcm2 ZnF of one MCM ring appears poised to partially occlude the N-terminal pore of the opposed MCM ring (Figure 5D). A comparison with the published structures shows that the Mcm2 in dCMGDo overlaps with the position occupied by duplex DNA in the double hexamer. Here, the same side of Mcm2 that touches the DNA backbone in the double hexamer,^6,7,30,31^ instead faces the opposed Mcm2 ZnF across dCMGDo (Figure S6A). In summary, double hexamer to dCMGDo transition triggers a rotation of the two MCM rings so that their central pores become misaligned. This causes each Mcm2 ZnF to plug the opposed MCM ring pore so that a DNA interacting element in Mcm2 becomes occluded. These changes upon dCMGDo formation would make it impossible for DNA to thread through the juxtaposed N-terminal domains of MCM while maintaining a double helical character. The clockwise rotation of two MCM rings of the double hexamer observed in the dCMGDo, and the prediction that duplex DNA is untwisted at the dimerization interface, matches a hypothetical fork-establishment mechanism by Eric Enemark.^49^ This mechanism was shown using an ingenious physical model for double hexamer separation, built with wood sticks and strings. In this model, the two DNA strands were pulled on to mimic 3^0^ to 5^0^ single-stranded DNA spooling established for CMG and the two MCM rings were allowed to pivot around one MCM hinge dimer across the double hexamer. As a result, the opposed side of MCM (for us, Mcm7) swings out, and the DNA at the N-terminal dimerization interface is unwound and sheared apart (Figure 5A). This strikingly matches our conformational transitions modeled after the dCMGDo structure.

It should be noted that the dCMGDo complex was released from chromatin via DNA digestion, and we considered the possibility that retaining DNA could limit the ability of two MCMs to become offset as observed in our structure. We note however that Mcm3 and Sld5 interactions with dimeric DONSON, essential for replication, can only be established if one MCM swings out. Instead, due to steric clashes, the DONSON dimer could not be engaged if two CMGs maintained a double hexamer inter-ring register (Figure S6B). Likewise, DONSON dimer engagement would not be possible if two CMGs interacted as observed in the yeast dCMG-Pol epsilon structure, as DONSON binding to one CMG complex would be too far to reach the Mcm3 A-domain and Sld5 B-domain of the second CMG (Figure S6C).

### The role of dimeric DONSON

**Figure 6:**
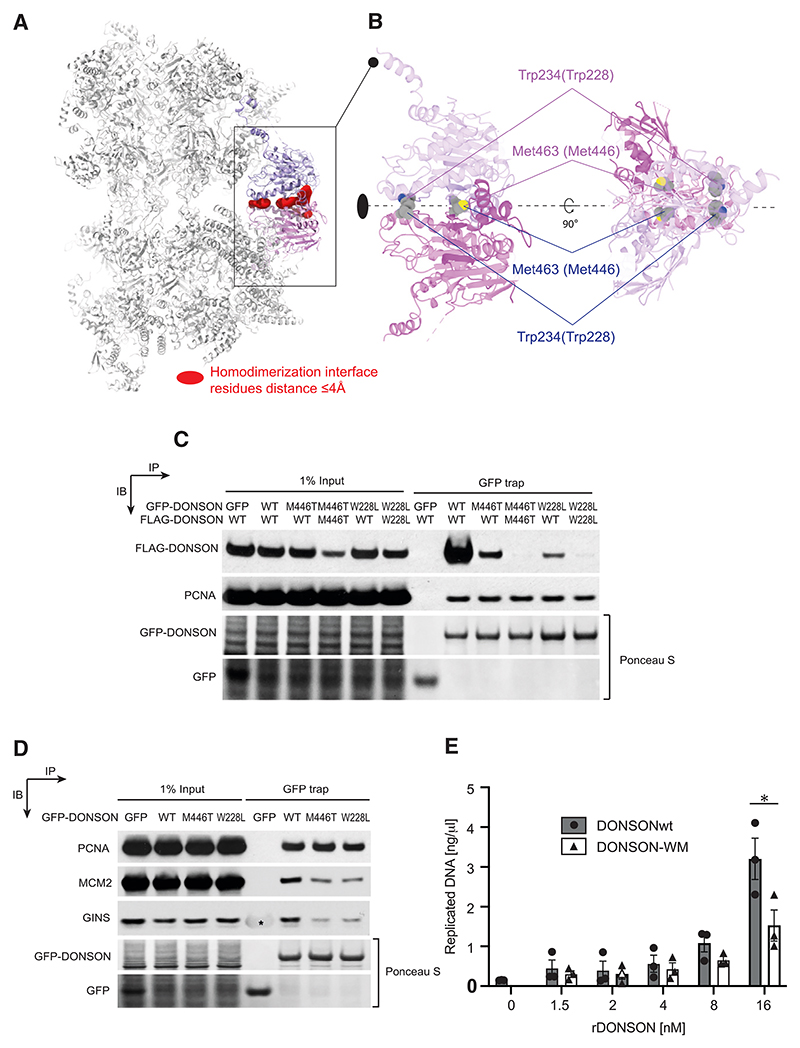
The role of DONSON dimerization in replication initiation (A) CMG dimerization through the DONSON dimer. Red surface identified distances of 4 Å or less be-tween surface residues of one monomeric CMG-DONSON subcomplex with the symmetry-related subcomplex. The only direct contacts keeping the dCMGDo complex together occur through dimerizing DONSON subunits. (B) Two views of DONSON reveal that residues Trp234 (human Trp228 mutated in patients) and Met463 (human Met446 mutated in patients) map at the homodimerization interface. (C) The patient-associated DONSON Met446Thr and Trp228Leu mutations disrupt dimerization. Cells were transfected with different combinations of WT and mutant DONSON tagged with either GFP or FLAG (as indicated). GFP-DONSON was purified from cell extracts using GFP-Trap and co-purified proteins were subjected to SDS-PAGE and western blotting with the antibodies indicated. Ponceau S stain was used to visualize the affinity purified GFP-tagged DONSON on the nitrocellulose filter. (D) The DONSON Met446Thr and Trp228Leu mutations disrupt binding to the GINS and MCM complex but not PCNA. Cells were transfected with WT and mutant DONSON tagged with GFP (as indicated). GFP-DONSON was purified from cell extracts using GFP-Trap and co-purified proteins were subjected to SDS-PAGE and western blotting with the antibodies indicated. Ponceau S stain was used to visualize the affinity purified GFP-tagged DONSON on the nitrocellulose filter. (*) The anti-body raised to the entire GINS complex cross-reacts with purified GFP protein. (E) DNA replication assay exploring the ability of a WM variant to rescue DNA replication. The replication reaction was set up in DONSON-depleted extract optionally supplemented with indicated concentrations of recombinant wild-type DONSON or DONSON WM mutant. Synthesis of nascent DNA was followed by incorporation of a^32^P-dATP into newly synthesized DNA in early S-phase. n = 3, mean with SEM presented; paired, nonparametric, two-tailed t test DONSON WT vs. DONSON WM p = 0.0312.

Inspection of the dCMGDo structure established that a unique reconfiguration of the double hexamer appears to be captured by (or imparted upon) the engagement of dimeric DONSON. At least for the globular core of the protein, each protomer in the DONSON dimer contacts one CMG complex alone, rather than bridging across the two helicases (Figure 6A). Hence, stability of the whole dCMGDo structure, featuring two offset MCM rings, appears dependent on DONSON homodimerization. We looked for amino acid changes that would disrupt dimer formation in our structure and came to realize that two residues, whose human counterparts are mutated in patients, map at the dimer interface in our structure (Figure 6B). One is Met463, which maps at the center of a hydrophobic pocket and makes direct contact with dimerization surface residues in cis. It corresponds to Met446 in humans, whose mutation to Thr is recessive and causes primordial dwarfism in patients.^43,50^ The second is Trp234, engaged in a hydrophobic interaction with equivalent Trp234 from the opposed protomer. It corresponds to Trp228 in humans, whose mutation to Leu is dominant negative, and was identified in a patient clinically diagnosed with Femoral-Facial syndrome.^51^ We created a human cell line containing GFP-tagged and FLAG-tagged DONSON either WT or containing the Met446Thr or Trp228Leu amino acid changes. The cell lines prepared were either expressing both tagged proteins as mutants (homozygous) or one WT and one mutant (heterozygous). We used western blotting with anti-FLAG M2 antibodies to test for protein expression levels and found a minor reduction in homozygous DONSON Trp228Leu compared with homozygous WT DONSON and a more significant decrease in Met446Thr expression, although the signal remained robust. We then immunoprecipitated DONSON using GFP trap and probed for dimer formation with the M2 antibody that would recognize FLAG-tagged DONSON. We found that heterozygous cells containing WT and Met446Thr alleles or WT and Trp228Leu alleles were significantly defective for DONSON dimer formation. This effect was exacerbated when using homozygous Met446Thr or Trp228Leu DONSON cell lines, with dimer formation completely impaired (Figure 6C). We then asked whether dimer disruption observed with patients’ mutations had any effect on DONSON’s ability to interact with the established replisome (proliferating cell nuclear antigen [PCNA]), GINS, or with MCM. Although PCNA interaction was unperturbed with all mutants tested, we found that both DONSON M446T and W228L variants have a defect in both GINS and MCM binding (Figure 6D). Finally, we wanted to test whether changes in the *Xenopus* proteins that recapitulate homologous patients’ mutations have any effect on DNA replication. To this end, we used bacterial expression to produce a recombinant DONSON variant (DONSON WM) containing both Met463 and Trp234 mutations. We used mass photometry to show that this variant presented defects in homodimerization (Figure S7). Indeed, although the WT protein formed homodimers in a range of protein concentrations, the DONSON WM’s ability to homodimerize was concentration dependent. We observed that although DONSON WM measured at 20 nM mainly formed homodimers, it was primarily monomeric at 5 nM (the concentration used to measure all other DONSON mutants). Given this observation, we decided to titrate WT and DONSON WM mutant in DNA replication experiments after immunodepletion of endogenous DONSON and measure nascent DNA synthesis in early S-phase. Although little DNA synthesis can be observed in the 1.5–4 nM concentration range, we observed that WT DONSON is more efficient at rescuing replication at 8 and 16 nM concentration (Figure 6E). Collectively, our data provide a link between primordial dwarfism, DONSON’s homodimerization and the ability to incorporate GINS into the CMG helicase, in a key step on the path to replication initiation.

## Discussion

We used the *Xenopus* egg extract system to isolate and image structural intermediates of replication initiation with cryo-EM. We employed conditions to activate origins but halt DNA synthesis. By doing this, we captured an initiating replisome assembly that is specific to higher eukaryotes. It contains DONSON, a replication factor that is absent in yeast and has recently been established as the assembly factor that incorporates GINS into the CMG complex.^41,42^ We used structural and biochemical methods to show that DONSON assembles as a homodimer, and we identified essential elements that support its engagement to both MCM and GINS. These structural elements map within flexible loops that emanate from the dimerization core of DONSON. We also identified interaction interfaces that involve the DONSON dimer core and structured domains in both GINS and MCM. These interactions however play a less important role in GINS recruitment and origin activation.

Our observations raise the question of whether DONSON homodimerization is at all important for GINS incorporation into the CMG. To address this question, we turned to cell lines of patients affected by primordial dwarfism. We identified two patients’ mutations resulting in amino acid changes at the DONSON dimer interface, and we discovered that dimerization of these protein variants is defective. The same dimerization defect was observed using recombinant expression of a *Xenopus* DONSON variant that contains the homologous amino acid changes. As primordial dwarfism is often associated with mutations in proteins involved in replication initiation,^52^ our findings support a role for DONSON dimerization in origin activation. To corroborate this notion, we asked whether, in human cells, the DONSON variants defective for dimerization are still able to interact with GINS and MCM, as observed in our structure. We found that interaction with both GINS and MCM is partially impaired, indicating a direct role for DONSON homodimerization in efficient CMG formation. We then used *Xenopus* egg extracts to show that interfering with DONSON homodimerization makes DNA replication rescue inefficient. Our data invite a model whereby dimeric DONSON recruits two GINS molecules to MCM concurrently, which matches our structural observation of a symmetric dCMGDo initiation intermediate. This mechanism mirrors previous findings with yeast proteins, suggesting that conversion from an inactive MCM double hexamer to a DNA melting CMG-Pol epsilon homodimer occurs symmetrically. Future structural work will establish whether homodimerization is also used by yeast Sld2 as a mechanism to recruit two GINS factors to the double hexamer on the path to origin DNA melting.

**Figure 7:**
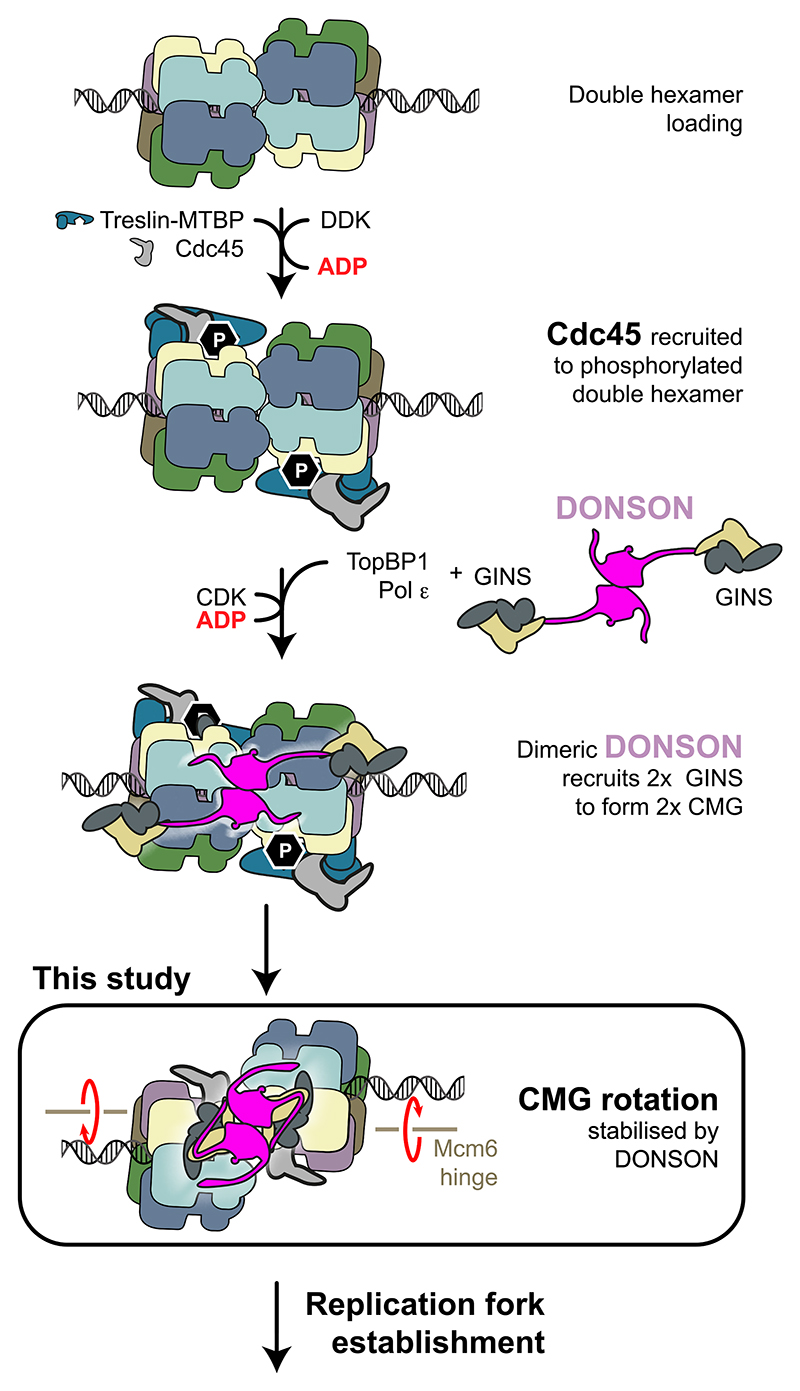
A model for the incorporation of two GINS factors into two CMG assemblies, mediated by dimeric DONSON Loaded MCM double hexamers are phosphorylated by DDK. The phosphorylation is read by Treslin-MTBP, which in turn promote Cdc45 recruitment onto the MCM double hexamer. A DONSON dimer engages two copies of the GINS firing factors. It recruits them to the MCM double hexamer by engaging the Mcm3 ATPase via the D3 loop. The DONSON dimer then locks onto the double hexamer, delivering GINS to form the CMG. This leads to a reconfiguration in the double hexamer structure, with two MCM rings that rotate clockwise with respect to one another and become offset ready for replication fork establishment.

What could be the structural mechanism whereby DONSON brings two GINS to assemble the dCMGDo? And how could this molecular intermediate mature to establish two diverging replication forks? Direct docking of DONSON to the MCM double hexamer, as seen in our structure, is impossible due to steric clashes. Thus, based on our functional and structural evidence, we propose that in the first holo-helicase assembly step, unstructured elements of dimeric DONSON, engaging Sld5 and the Mcm3 ATPase, support the incorporation of two copies of GINS into the two CMGs. We envisage that CMG assembly alone would lead to the rotation of two MCMs rings and nucleation of DNA unwinding inside of the two ATPase rings, similar to what we previously observed for the double CMG complex with yeast proteins.^27^ After this stage in origin activation, the two helicases must cross paths, so that the N-terminal dimerization interface of the double hexamer becomes poised at the front of the advancing replication fork.^25,28,29^ With our structure of a *Xenopus* dCMGDo activation intermediate, we established that the globular DONSON dimer captures the two helicases in a fixed relative orientation where the two MCM hexamers are no longer co-axial. The only way for this structure to exist on DNA involves the unwinding of the Watson and Crick strands at the dimerization interface^49^ (Figure 7). What happens after dCMGDo formation is more speculative. For example, lagging strand ejection could occur after dCMGDo formation with the two MCM ring pores misaligned. This step in helicase activation might also involve the poorly defined function of RecQL4 at initiation, which is known to act after CMG formation.^35–40^ Exposure of the lagging strand template would in turn trigger the recruitment of single-stranded DNA interacting factors Mcm10, RPA, and Pol alpha. Recruitment of these elongation factors could prompt the release of DONSON, which would in turn let go of the two CMG assemblies. The two helicases at this point would be free to continue along their trajectory toward replication fork establishment. In support of this model, the recent structure of Pol alpha bound to CMG shows that the primase subunit Pri2 engages the A-domain of Mcm3,^53^ on a surface that overlaps with the DONSON docking site (Figure S6D). Thus, Pol alpha recruitment to the CMG could compete out dimeric DONSON, and its release from origins could finalize dCMG separation. A DONSON dimer could either remain tethered via a flexible element to one of the two diverging CMGs or be recruited to the advancing replisome at a later stage, when its fork protection functions are required. In this context, it is possible that robust DONSON engagement to a single CMG, similar to what was observed in our structure, will selectively occur at a stalled replication fork, when Okazaki fragments are not being synthesized.

Our previous work established that single-particle cryo-EM can be used to visualize intermediates in replication origin activation reactions, reconstituted *in vitro* with purified yeast protein.^3,17,27^ With that approach, we used biochemical tools to stage helicase activation, for example by omitting the essential yeast Mcm10 factor that activates DNA unwinding on the path to origin activation. Our current work extends our structural analysis in three ways. First, using the *Xenopus* egg extract, we analyze the full complexity of the cellular replication process. Second, we introduced purification approaches to target our structural analysis to the CMG replicative helicase assembly and ignore licensed origins that have not started the initiation process. This reduces sample heterogeneity to levels manageable with single-particle cryo-EM. Third, by using an ATM/ATR inhibitor and a replicative-polymerase inhibitor, we showed that we could control intersecting pathways to ask detailed questions regarding structural mechanism. In future work, we aim to image replication reactions in the context of chromatin.

### Limitations of the study

Three items limit our study. First, the dCMGDo complex was purified after digesting chromatin with benzonase, and hence, it does not contain DNA. Spatial constraints imposed by symmetric binding of functionally important DONSON elements imply that the relative orientation of the two CMGs is unlikely to change when DNA is retained inside the protein complex. That said, the presence of DNA will likely impart changes to the ATPase tier, including changes in nucleotide occupancy. Imaging the dCMGDo complex on DNA is our future aim. Second, we used aphidicolin to block replicative polymerase activity. Although this strategy captures an early replication intermediate, it does not allow for performing meaningful kinetic studies on the dCMGDo, following complex formation and separation as DNA replication progresses. Third, it remains important to address whether competitive MCM binding by Pol alpha and DONSON has a role in initiating DNA replication.

While our manuscript was under review, two articles were published, predicting^54^ or describing^55^ the structure of monomeric CMG bound to DONSON. These studies agree with our structure-function characterization and with articles cited in our paper.^41,42^ A prediction that two assembled helicases rotate to achieve full DONSON engagement^54^ matches our structural observations. Possible competitive CMG binding for DONSON and Pol alpha in regulating fork establishment and progression is also noted.^55^

## Star*Methods

### Key Resources Table

**Table:** 

REAGENT or RESOURCE	SOURCE	IDENTIFIER
Antibodies		
Sheep polyclonal anti-Psf2	Gambus lab	Gambus et al.^56^
Sheep polyclonal anti-Cdc45	Gambus lab	Gambus et al.^56^
Sheep polyclonal anti-GINS	Gambus lab	Tarcan et al.^57^
Rabbit polyclonal anti-DONSON	Gambus lab	Kingsley et al.^42^
Sheep polyclonal anti-DONSON	Gambus lab	Kingsley et al.^42^
Sheep polyclonal anti-Mcm7	Gambus lab	Priego Moreno et al.^58^
Sheep polyclonal anti-RecQ4	Gambus lab	Moreno et al.^59^
Sheep polyclonal anti-TopBP1	Gambus lab	Moreno et al.^59^
Sheep polyclonal anti-Treslin	Blow lab	Volpi et al.^60^
Sheep polyclonal anti-MTBP	Blow lab	Volpi et al.^60^
IgG from rabbit serum	Sigma-Aldrich	RRID: AB_1163659
Polyclonal rabbit anti-FLAG	Sigma-Aldrich	RRID: AB_439687
Monoclonal mouse anti-Mcm2	BD Transduction labs	RRID: AB_2141952
Monoclonal mouse anti-PCNA	Santa Cruz Biotechnology	RRID: AB_628110
Monoclonal mouse anti-PCNA	Sigma Aldrich	RRID: AB_477413
Bacterial and virus strains		
Rosetta™(DE3) pLysS Competent Cells	Novagen (Merck)	70956
BL21(DE3) Competent Cells	Novagen (Merck)	69451
Chemicals, peptides, and recombinant proteins		
Aphidicolin	Bio-Techne Limited	Cat# 5736/1
Caffeine	Sigma-Aldrich	CAS: 58-08-2
ANTI-FLAG® M2 Magnetic Beads	Sigma-Aldrich	Cat# M8823
3X FLAG Peptide	Stratech Scientific Ltd	A6001
HisTrap HP	Cytiva	Cat# 17524801
HiTrap Heparin HP	Cytiva	Cat# 17040701
Cdc45-TEV-His_10_-FLAG5	This study	Addgene agreement 83268
DONSON WT	Gambus lab	Kingsley et al.^42^
DONSON DEW (D374A; E377A; W381A)	This study	Addgene agreement 83268
DONSON S437A	This study	Addgene agreement 83268
DONSON WM (W234L; M463T)	This study	Addgene agreement 83268
DONSON 3A (V473A; R476A; Y481A)	This study	Addgene agreement 83268
DONSON D24 (D1-24)	This study	Addgene agreement 83268
DONSON DEWS (D374A; E377A; W381A; S437A)	This study	Addgene agreement 83268
DONSON DD1 (D1-155)	Gambus lab	Kingsley et al.^42^
GINS: (Psf1, Psf2, Psf3 and Sld5)	Gambus lab	Tarcan et al.^57^
Graphene oxide	Sigma-Aldrich	763705-25ML
Deposited data		
*Xenla* dCMGDo consensus cryo-EM map	This study	EMDB: EMD-18191
*Xenla* dCMGDo ATPase cryo-EM map	This study	EMDB: EMD-18192
*Xenla* sCMG consensus cryo-EM map	This study	EMDB: EMD-18195
*Xenla* dCMGDo – without MCM ATPase PDB coordinates	This study	PDB: 8Q6O
*Xenla* dCMGDo – MCM ATPase PDB coordinates	This study	PDB: 8Q6P
*Xenopus laevis Cdc45-FLAG interactions on chromatin during DNA replication – Mass spectrometry data project*	This study	PRIDE: PXD044422
Experimental models: Cell lines		
293FT	Thermo Fisher Scientific	R70007
Experimental models: Organisms/strains		
*Xenopus laevis*	Gambus lab	N/A
Oligonucleotides		
DONSON DEW (D374A; E377A; W381A) forward: CTCTGCGCTAGAAGAGATGGGAGTTGAAGA TAAAATTAAAAAG;	Sigma-Aldrich	N/A
DONSON DEW (D374A; E377A; W381A) reverse: AAGCTCGCATCTTCCGCGCTTTCATC CGATGCCTC	Sigma-Aldrich	N/A
DONSON S437A forward: AAACTGTAAGGC GATTGTCGCTGC	Sigma-Aldrich	N/A
DONSON S437A reverse: TGAGGAAGTT CAGTAAAG	Sigma-Aldrich	N/A
DONSON W234L forward: GTCGCTTCCTCT GGTACAGCTTTTTC	Sigma-Aldrich	N/A
DONSON W234L reverse: GGATGAATCCAG TGCACAAG	Sigma-Aldrich	N/A
DONSON M463T forward: AGGAGCAACAA CGCACGCGCTTA	Sigma-Aldrich	N/A
DONSON M463T reverse: CGGAATGCGA CTGGAGAC	Sigma-Aldrich	N/A
DONSON 3A (V473A; R476A; Y481A) forward: GAACTCTGGTGCGAAGGATCAGTTTAGTCTGG;	Sigma-Aldrich	N/A
DONSON 3A (V473A; R476A; Y481A) reverse: ACCGCAGTCTTCGCATTCACACTCCTTG CCTTAAG	Sigma-Aldrich	N/A
DONSON D24 (Δ1-24) forward: AGGAGCAGG AGCGAGGCT	Sigma-Aldrich	N/A
DONSON D24 (Δ1-24) reverse: CAGATCCTCT TCTGAGATGAGTTTTTGTTCG	Sigma-Aldrich	N/A
DONSON DD1 (Δ1-155) forward: TCCGTGAC TTTTCCTGCT	Sigma-Aldrich	N/A
DONSON DD1 (Δ1-155) reverse: CATTTATTT CCAGGTGTACC	Sigma-Aldrich	N/A
Recombinant DNA		
pET28a-DONSON	Gambus lab	Addgene: 208797
pET28a-DONSON ΔD1	Gambus lab	Addgene: 208804
pET28a-DONSON-WM (W234L, M463T)	This study	Addgene: 208798
pET28a-DONSON-DEWS (D374A, E377A, W381A, S437A)	This study	Addgene: 208799
pET28a-DONSON-3A (V473A, R476A, Y481A)	This study	Addgene: 208800
pET28a-DONSON Δ24 (1-24 removed)	This study	Addgene: 208801
pET28a-DONSON-S437A	This study	Addgene: 208803
pET28a-DONSON-DEW (D374A, E377A, W381A)	This study	Addgene: 208802
Software and algorithms		
crYOLO 1.4.0	Wagner et al.^61^	https://cryolo.readthedocs.io/en/stable/
Relion 3.0.4 and 4.0.0	Kimanius et al.^62;^ Zivanov et al^63^	https://www.mrc-lmb.cam.ac.Uk/relion/index.php?title=Main_Page
cryoSPARC v4.2.1	Punjani et al.^64^	https://cryosparc.com/
Gctf	Zhang et al.^65^	https://sbgrid.org/software/titles/gctf
CtfFind-4.1	Rohou et al.^66^	https://grigoriefflab.umassmed.edu/ctffind4
Topaz-0.2.5	Bepler et al.^67^	https://cb.csail.mit.edu/cb/topaz/
Coot V0.9.8.1	Emsley et al.^68^	https://www.mrc-lmb.cam.ac.uk/personal/pemsley/coot/
PHENIX V1.20.1	Liebschner et al.^69^	http://www.phenix-online.org/
UCSF Chimera V1.16	Pettersen et al.^70^	https://www.cgl.ucsf.edu/chimera/
UCSF ChimeraX	Goddard et al.^71^	https://www.rbvi.ucsf.edu/chimerax/
Mascot v 2.8.0.1	Matrix Science, London, UK	https://www.matrixscience.com/
Scaffold v 5.1.0	Proteome Software Inc., Portland	https://proteomesoftware.com/
AcquireMP v 2.5	Refeyn Ltd., Oxford, UK	https://www.refeyn.com/
DiscoverMP v 2.5	Refeyn Ltd., Oxford, UK	https://www.refeyn.com/
GraphPad Prism v 10.0.2	GraphPad Software LLC	https://www.graphpad.com/

## Resource Availability

### Lead contact

Further Information and requests for resources and reagents should be directed to and will be fulfilled by the lead contact, Alessandro Costa (alessandro.costa@crick.ac.uk).

### Materials availability

Plasmids generated in this study are deposited to Addgene with accession numbers indicated in the key resources table.

### Data and code availability

- Datasets are available as indicated below:
- Mass spectrometry analysis of immunoprecipitated Cdc45-TEV-His_10_-FLAG_5_ were deposited in PRIDE database and publicly available as of the date of publication. Accession numbers are listed here and in the key resources table. Project Name: *Xenopus laevis* Cdc45-FLAG interactions on chromatin during DNA replication. Project accession: PRIDE: PXD044422 Project https://doi.org/10.6019/PXD044422x
- Cryo-EM density maps used in model building have been deposited in the Electron Microscopy Data Bank (EMDB) https://www.ebi.ac.uk/pdbe/emdb, under the following accession numbers: EMDB: EMD-18195 for *Xenla* sCMG consensus, EMDB: EMD-18191 for *Xenla* dCMGDo consensus, EMDB: EMD-18192 for *Xenla* dCMGDo MCM ATPase.
- Atomic coordinates have been deposited in the Protein Data Bank (PDB), http://www.pdb.org, with the following accession numbers: PDB: 8Q6O for *Xenla* dCMGDo without ATPase, PDB: 8Q6P for *Xenla* dCMGDo MCM ATPase.
- All original western blots are available at: Mendeley Data:1 The full mass spectrometry data for Figure 1 is deposited in PRIDE: PXD044422.
- This paper does not report original code.
- Any additional information required to reanalyse the data reported in this paper is available from the lead contact upon request.

## Experimental Model and Study Participant Details

### *Xenopus laevis* egg extract model system

African clawed toads (*Xenopus laevis*) were maintained in Birmingham animal house. The animals are housed five per tank at 20°C and fed five times a week. For preparation of egg extracts only fully grown females were used as they are the only sex able to lay eggs. For preparation of demembranated sperm, only mature males were used.

All the work with *Xenopus laevis* was approved by Animal Welfare and Ethical Review Body (AWERB) at University of Birmingham and approved by UK Home Office in the form of the Project License issued for Dr Agnieszka Gambus.

All experiments performed conformed to the UK Home Office standards.

### Cell lines

293FT (Thermo Fisher Scientific) cells were routinely grown in DMEM supplemented with 10% FCS, 5% L-glutamine and 5% penicillin-streptomycin. The cell line was routinely tested for mycoplasma by PCR.

### *Escherichia coli* strains

For recombinant expression of DONSON variants, BL21 (DE3) strain was used. Cells were grown in 2 L of LB media. After cells reached OD_600_=0.6, 1 mM IPTG was added, followed by overnight incubation at 18°C.

Cdc45-TEV-His_10_-FLAG_5_ was expressed in Rosetta (DE3) pLysS competent cells (Novagen, Merck Millipore). Bacteria were grown in auto inducing media (Formedium) at 37°C until OD_600_=0.6, then moved to 18°C overnight.

### Experimental source material for *in vitro* work

The source organism of recombinant proteins expressed in bacteria and analysed was *Xenopus laevis*.

## Method Details

### *Xenopus laevis* egg extract

*Xenopus laevis* egg extracts were prepared as previously described.^72^ Metaphase II arrested *X. laevis* egg extract is made from unfertilised female frog oocytes. In order to increase the amount of stage six (mature) oocytes, ten frogs were primed with 150 units follicle stimulating hormone Foligon (Intervet) two to seven days before eggs were required. The day before the eggs collection, frogs were injected with 400-600 units of serum gonadotropin Chorulon (Intervet), and subsequently transferred to laying tanks containing 2.5 L of 1x MMR (0.1 M NaCl, 2 mM KCl, 1 mM MgCl_2_, 2 mM CaCl_2_, 0.1 mM EDTA, 5 mM HEPES, pH to 7.8 with NaOH). Frogs were kept in the tanks laying eggs overnight at ≤ 23°C. Eggs from different frogs were collected the next morning in a 1 L glass beaker. Apoptotic and immature eggs were not collected. Eggs were rinsed with 1x MMR, and most of the buffer was subsequently removed. Eggs were then de-jellied by rinsing them in cysteine solution (2.2% cysteine, 5mM EGTA, pH to 7.6 with KOH). De-jellied eggs were rinsed again with 1x MMR, and then washed in UEB buffer (50 mM KCl, 50 mM HEPES, 5 mM MgCl_2_, 5 mM EGTA, 2 mM DTT, pH to 7.6 with KOH) and white/swollen apoptotic eggs floating on the top were removed with a Pasteur pipette. The de-jellied eggs were packed into 14 ml round bottom polypropylene tubes (187261; Greiner) with 1 ml UEB containing 10 µg/ml protease inhibitors: aprotinin, leupeptin and pepstatin and 50 µg/ml Cytochalasin D (C8273-5MG, Sigma) and the excess buffer from the settling eggs was removed. The tubes were then spun to pack the eggs in a Beckman JS13 rotor at 800 g for 1 min, room temperature (RT). The white apoptotic swollen eggs that float to the top were removed using a Pasteur pipette followed by another centrifugation at high speed at 10,000 g for 10 min at RT. This results in separation of the eggs into a lipid layer at the top, brown cytoplasmic fraction in the middle and an insoluble egg yolk pellet at the bottom. The cytoplasmic layer was collected using a 20 G needle and a 1 ml syringe via side puncture and from this point the extract was kept on ice. Extract was supplemented with 10 µg/ml protease inhibitors, 10 µg/ml Cyto-chalasin D and 15% of LFB1/50 (10% sucrose, 50 mM KCl, 40 mM HEPES pH 8, 20 mM K phosphate pH 8, 2 mM MgCl_2_, 1 mM EGTA, 2 mM DTT, 1 µg/ml of each: aprotinin, leupeptin and pepstatin). The extract was gently and well mixed using a Pasteur pipette and transferred to SW55 ultracentrifuge tubes (344058, Beckmann) which were then subjected to a final clarifying spin at 30,000 g for 20 min at 4°C. After the spinning, the yellow lipid plug from the top was removed with a clean spatula, and the pale yellow cytoplasmic fraction collected, taking extra care to not disturb the layer bellow it containing mitochondria (mitochondria will lyse after freezing-thawing the extract and will promote apoptosis rendering the extract useless). The collected extract was then supplemented with 1% v:v of glycerol, and frozen in liquid nitrogen in small beads by pipetting it dropwise and stored at -80°C.

### Demembranated *Xenopus* sperm

*Xenopus laevis* demembranated sperm was prepared as previously described.^72^ 15 male frogs were euthanised by placing them into individual chambers containing MS222 anaesthetic solution. Testes were then localised in the lower abdominal region and carefully cut out and placed in EB solution (50 mM KCl, 5 mM MgCl_2_,2 mM β-mercaptoethanol, 50 mM HEPES-KOH, pH 7.6) on ice. Testes were then washed with approximately 20 ml of EB solution in a 9 cm Petri dish, and cleaned by carefully removing any blood vessels and extraneous tissue using dissection forceps. Cleaned testes were then transferred to a clean 9 cm Petri dish containing 2 ml EB solution, and chopped as finely as possible with a razor blade. Chopped material was kept on ice, and once all testes were chopped, the resultant mixture was filtered through a 25 mm mesh nylon filter mounted on a small funnel. The filtered material was then transferred to a 15 ml Falcon tube and centrifuged at 2,000 g in a swinging bucket rotor for 5 minutes at 4°C. Pelleted sperm nuclei were resuspended in 0.5 ml of SuNaSp per testis (0.25 M sucrose, 75 mM NaCl, 0.5 mM spermidine, 0.15 mM spermine, 15 mM HEPES-KOH pH 7.6) and subsequently supplemented with 25 µl of lysolecithin (5 mg/ml) per testis and incubated for 5 min at RT. Demembranation of the sperm was determined by mixing 1 µl of the sperm sample with 1 µl of Hoechst 33258 (20 µg/ml) and imaged by UV microscopy. Demembranated sperm stained bright blue with Hoechst, while non demembranated sperm did not. If less than 95% of the sperm was demembranated, the mixture was re-spun, pellet resuspended in fresh SuNaSp and lysolecithin treatment repeated as before. Once demembranated, the homogenate was centrifuged at 2,000 g in a swinging bucket rotor for 5 minutes at 4°C, and lysolecithin was quenched by resuspending the pellet in 0.5 ml per testis of SuNaSp containing 3% BSA. Demembranated sperm nuclei were washed twice with EB solution. After the second wash, the pellet was resuspended in 100 µl per testis of EB containing 30% glycerol. The sperm DNA concentration in the final mixture was estimated by counting the number of sperm using a hemocytometer (counting repeated four times). Somatic-type nuclei were counted as two sperm nuclei as they are diploid. Given that the *Xenopus* haploid genome corresponds to 3 pg of DNA, concentration of DNA in the homogenate could be estimated after counting the number of haploid nuclei. Finally, demembranated sperm nuclei were aliquoted into 80 µl fractions and stored at -80°C

### DNA synthesis assay

Interphase *Xenopus laevis* egg extract was supplemented with 10 ng/µl of demembranated sperm chromatin and incubated at 23°C for indicated time. Synthesis of nascent DNA was then measured by quantification of α^32^P-dATP (PerkinElmer) incorporation into newly synthesised DNA, as described before.^72^ The extract contains endogenous dNTP pools of ~ 50 µM.^73^ The total amount of DNA synthesized, expressed as ng DNA/µl extract, can then be calculated by multiplying percent total ^32^P incorporated by a factor of 0.654.^73^ This calculation assumes an average molecular weight of 327 Da for dNMPs and equal quantities of all four dNTPs incorporated into DNA (weight of dNMP incorporated in ng/µl = percent total ^32^P incorporated/100 × 50 × 10^6^ × 4 × 327 × 10^3^).^73^

### Egg extract immunodepletion

DONSON immunodepletions with rabbit antibodies were performed using Dynabeads Protein A (10008D, Life Technologies) coupled to *Xenopus* DONSON antibodies raised in rabbit and affinity purified, or nonspecific rabbit IgG (I5006, Sigma-Aldrich). The DONSON antibodies were coupled at 600 µg per 1 ml of beads. Effective immunodepletion required two rounds of 45 min incubation of egg extract with antibody coupled beads at 50% beads ratio.

Cdc45 immunodepletions were performed using Dynabeads Protein A (10008D, Life Technologies) coupled to Cdc45 antibodies raised in rabbit and affinity purified, or nonspecific rabbit IgG (I5006, Sigma). The Cdc45 antibodies were coupled at 600 µg per 1 ml of beads and incubated with 660 µl of extract. Complete immunodepletion required three rounds of 20 min incubation at room temperature with the extract.

### Chromatin isolation time course

Interphase *Xenopus laevis* egg extract was supplemented with 10 ng/µl of demembranated sperm DNA and subjected to indicated treatments. The reaction was incubated at 23°C for indicated length of time and chromatin was isolated in ANIB100 buffer (50 mM HEPES pH 7.6, 100 mM KOAc, 10 mM Mg(OAc)_2_, 2.5 mM Mg-ATP, 0.5 mM spermidine, 0.3 mM spermine, 1 µg/ml of each aprotinin, leupeptin and pepstatin, 25 mM β-glycerophosphate and 10 mM 2-chloroacetamide (Merck)) as described previously.^72^ During the chromatin isolation procedure, a sample without addition of sperm DNA (no DNA) is processed in an analogous way, usually at the end of the time course, to serve as a chromatin specificity control. The bottom of the PAGE gel on which the chromatin samples were resolved is cut off and stained with Colloidal Coomassie (SimplyBlue, Life Technologies) to stain histones which provide loading controls and indications of sample contamination with egg extract (cytoplasm). Antibodies used: anti-PCNA (Sigma Aldrich, P8825), anti-MCM2 (BD Transduction labs, 610700), anti-FLAG (Sigma Aldrich, F7425), anti-Psf2 and anti-Cdc45,^56^ anti-GINS,^57^ sheep anti-DONSON and rabbit anti-DONSON,^42^ anti-Mcm7,^58^ anti-RecQ4 and anti-TopBP1,^59^ anti-Treslin and anti-TopBP1.^60^

### Recombinant protein purification

*Xenopus laevis* Cdc45 gene was cloned in a bacterial pRSFDuet-1 T7 expression plasmid (Novagen) fused to a C-terminal, TEV-cleavable-His_10_-FLAG tag and codon optimized for expression in *E. coli* by Pellegrini’s lab.^74^ The vector was modified by adding further four FLAG tags via PCR to have *Xenopus laevis* Cdc45 fused with a TEV-cleavable-His_10_-FLAG_5_ tag.

Cdc45-TEV-His_10_-FLAG_5_ was expressed in Rosetta (DE3) pLysS competent cells (Novagen, Merck Millipore). Bacteria were grown in auto inducing media (Formedium) at 37°C until OD=0.6, then moved to 18°C overnight. Bacteria were pelleted at 6000 g for 10 min at 4°C, resuspended in 50 mM Tris, 100 mM NaCl, 10% sucrose and frozen. Lysis was performed by bringing the suspension to 500 mM NaCl, 15 mM imidazole, 1 mM PMSF, 0.1% Triton X-100, 1 mg/ml lysozyme and adding one protease inhibitor cocktail tablet (Roche cOmplete), then freezing and thawing in liquid nitrogen three times followed by incubation for 1h at 4°C. The lysate was spun at 20,000 g for 30 min at 4°C and the soluble fraction incubated with 30 U/ml benzonase nuclease (E1014-25KU, Sigma) for 1 h at 4°C. Finally, the lysate was spun at 20,000 g for 30 min at 4°C and the soluble part collected.

Purifications were performed using an ÄKTAprime plus system. The lysate was loaded onto a nickel-affinity column (Cytiva HisTrap HP) equilibrated in His binding buffer (50 mM Tris pH 7.5, 500 mM NaCl and 0.1 mM PMSF) and Cdc45-TEV-His_10_-FLAG_5_ was eluted with an imidazole gradient of 3-80% over 100 ml at 1 ml/min (His elution buffer: 50 mM Tris pH 7.5, 500 mM NaCl, 500 mM imidazole and 0.1 mM PMSF). The eluted fractions corresponding to the elution peak measured at 280 nm were collected and dialysed into Heparin binding buffer (50 mM Tris pH 7.5, 1 mM EDTA, 1 mM DTT and 0.1 mM PMSF) overnight. The protein was then loaded onto a heparin column (Cytiva HiTrap Heparin HP) equilibrated in Heparin binding buffer and eluted with a salt gradient of 5-40% over 100 ml at 1 ml/min (Hep elution buffer: 50 mM Tris pH 7.5, 2 M NaCl, 500 mM imidazole and 0.1 mM PMSF). The protein was then dialysed in LFB1/50 buffer (10% sucrose, 50 mM KCl, 40 mM HEPES pH 8.0, 20 mM K phosphate pH 8.0, 2 mM MgCl_2_, 1 mM EGTA, 2 mM DTT, 1 µg/ml of each: aprotinin, leupeptin, and pepstatin) and stored at -80°C at final concentration of 42 mM.

pET28a-DONSON, pET28a-DONSON-WM (W234L, M463T), pET28a-DONSON-DEWS (D374A, E377A, W381A, S437A), pET28a-DONSON-3A (V473A, R476A, Y481A), pET28a-DONSONΔ24 (1-24 removed), pET28a-DONSON-S437A, pET28a-DONSON-DEW (D374A, E377A, W381A) vectors were used for protein expression in 2L of BL21 (DE3) (1 mM IPTG was added at OD_600_ = 0.6, followed by incubation overnight at 18°C). Frozen bacterial pellets were lysed in resuspension buffer (500mM NaCl, 50mM NaH2PO4, 2mM MgCl2, 10% glycerol, pH 9, 1 µg/ml of each: aprotinin, leupeptin and pepstatin) and supplemented with 1 mg/ml lysozyme, 0.05% Tween-20, and BitNuclease. After sonication, the lysate was clarified by centrifugation at 14,000 g for 30 min at 4°C, and supernatant fractions containing soluble proteins were then incubated with 2 ml of pre-washed Super Ni-NTA Affinity Resin (SUPER-NINTA100, Generon) for 2 hours rotating at 4°C. The beads were then washed three times with 30 ml of Lysis buffer, both supplemented with 15 mM imidazole. The beads were transferred to 10 ml columns (Poly-Prep Chromatography Column, Bio-Rad) and eluted with Lysis Buffer supplemented with 250 mM imidazole. Most concentrated elution fractions were dialysed against LFB1/50 buffer (10% sucrose, 50 mM KCl, 40 mM HEPES pH 8.0, 20 mM K phosphate pH 8, 2 mM MgCl_2_, 1 mM EGTA, 2 mM DTT, 1 µg/ml of each: aprotinin, leupeptin and pepstatin) and snap frozen in liquid nitrogen.

FLAG_5_, His_6_-tagged GINS was purified as described in Tarcan et al.^57^

### Large-scale immunoprecipitation from chromatin

4 ml of *Xenopus laevis* egg extract were activated into interphase by adding energy regenerator (50 mM HEPES pH 7.6, 306 mg/ml creatine phosphate, 3.6 mg/ml creatine phosphokinase), 0.25 mg/ml cycloheximide and 0.3 mM CaCl_2_ and supplemented with 10 ng/µl of demembranated sperm DNA, 70 nM recombinant Cdc45, 40 µM aphidicolin, 5 mM caffeine and incubated at 23°C for 60 min. Chromatin was isolated in ANIB100 buffer (50 mM HEPES pH 7.6, 100 mM KOAc, 10 mM Mg(OAc)_2_, 2.5 mM Mg-ATP, 0.5 mM spermidine, 0.3 mM spermine, 1 µg/ml of each aprotinin, leupeptin and pepstatin, 25 mM β-glycerophosphate, 0.1 mM Na_3_VO_4_ and 10 mM 2-chloroacetamide) as described previously.^72^

Chromatin pellets were resuspended in ANIB100 containing 20% sucrose. Protein complexes were released from chromatin by digestion with 0.4 U/µl benzonase nuclease (E1014-25KU, Sigma) and sonicated for 5 min using a sonicator with settings: 30 s on, 30 s off, low power (Bioruptor Diagenode UCD-200). Immunoprecipitation was performed using 100 µl of anti-FLAG M2 magnetic beads (Sigma-Aldrich). Before elution the sample was washed four times with 1 ml of ANIB100 20% sucrose, ANIB100 20% sucrose 0.1% Triton X-100, ANIB100 and elution buffer (25 mM HEPES pH 7.5, 100 mM KOAc, 5 mM Mg(OAc)_2_, 1 mM ATP and 0.02% NP-40), respectively. The sample was eluted adding 250 µM 3xFLAG peptide (Stratech) to 200 µl of elution buffer.

The immunoprecipitated sample was separated via SDS-PAGE (NuPAGE™4 to 12%, Bis-Tris, Invitrogen) and silver stained (SilverQuest™, Invitrogen).

### Mass spectrometry

Gel digests were analysed by nano LC/MS/MS with a Waters M-class HPLC system interfaced to a ThermoFisher Oribitrap Fusion Lumos. Peptides were loaded on a trapping column and eluted over a 75 µm analytical column at 350 nl/min; both columns were packed with Luna C18 resin (Phenomenex). A 30 min gradient was employed. The mass spectrometer was operated in data-dependent mode, with MS and MS/MS performed in the Orbitrap at 60,000 resolution and 15,000 resolution, respectively. Advanced Peak Detection was turned on. The instrument was run with a 3 s cycle for MS and MS/MS. Proteome Discoverer v1.4 was used for peak generation.

### Mass-spectrometry data processing

Data were searched using Mascot (Matrix Science, London, UK; version 2.8.0.1) with the following parameters: Enzyme: Trypsin Fully Specific

Database: Uniprot Xenopus (forward and reverse appended with common contaminants) released on 04/15/2014. 79,274 (including reverse and CON) entries in the database were searched.

Fixed modification: Carbamidomethyl (C)

Variable modifications: Oxidation (M), Acetyl (Protein N-term), Deamidation (NQ), GlyGly (K), Phospho (STY)

Mass values: Monoisotopic

Peptide Mass Tolerance: 10 ppm

Fragment Mass Tolerance: 0.02

Da Max Missed Cleavages: 2

Mascot DAT files were parsed into the Scaffold (version Scaffold_5.1.0, Proteome Software Inc., Portland, OR) software for validation, filtering and to create a non-redundant list per sample. Data were filtered with 1% protein and peptide false discovery rate (FDR) and requiring at least two unique peptides per protein.

Peptide identifications were accepted if they could be established at greater than 34.0% probability to achieve an FDR less than 1.0% by the Percolator posterior error probability calculation.^75^ Protein identifications were accepted if they could be established at greater than 99.0% probability to achieve an FDR less than 1.0% and contained at least two identified peptides. Protein probabilities were assigned by the Protein Prophet algorithm.^76^ Proteins that contained similar peptides and could not be differentiated based on MS/MS analysis alone were grouped to satisfy the principles of parsimony. For calculation of fold enrichment for proteins with no peptides detected in control immunoprecipitation, that number was changed to one to allow for fold enrichment calculation.

### GFP-Trap affinity purification from human cells

For GFP-Trap pulldown experiments with 293FT cells, confluent cells were transfected with 12 µg of plasmid DNA using Lipofectamine 2000 and harvested 48 h post-transfection. Cells were incubated in lysis buffer (150 mM NaCl, 50 mM Tris HCl pH 7.5, 2 mM MgCl_2_, 0.5-1% NP40, 250 U/ml Benzonase (Merck) and EDTA-free protease inhibitor cocktail [Roche]) for 30 min with rotation at 4°C. Cell lysates were then pre-cleared at 65,000 g at 4°C for 30 min. For GFP-Trap, 5-7 mg of lysate was incubated with GFP-Trap agarose beads (ChromoTek) for 5 h at 4°C. The resulting GFP-Trap complexes were washed with wash buffer (100-300 mM NaCl, 50 mM Tris HCl pH 7.5, 0.5% NP40, and complete protease inhibitor cocktail [Roche]) and analysed by SDS–PAGE.

Immunoblotting was performed with antibodies Flag (Sigma-Aldrich, F7425; 1:5000), GINS (Gambus lab; 1:500), MCM2 (BD Transduction labs, 610700; 1:2000), CDC45 (Cell Signaling Technology, 11881; 1:500), PCNA (Santa Cruz Biotechnology, sc-56; 1:1000), GFP (Abcam, ab6556; 1:2000) and TOPBP1 (Fortis Life Science, A300-111A; 1:500).

### Mass photometry

Solution-phase mass determination of native DONSON and mutants thereof were acquired using the TwoMP (Refeyn) mass photometer. Experimental data were obtained in the form of mass photometry videos recorded for one minute using the AcquireMP v2.5 software (Refeyn) on precleaned, high sensitivity microscope slides. Prior to analysis of target proteins, a mass calibration was determined using urease, BSA and aldolase as standards spanning a mass range of 66 to 544 kDa. Each standard was used at a final concentration of 2 to 5 nM by diluting ten-fold into 18 µl of Dulbecco PBS buffer. A linear mass calibration was obtained after fitting of Gaussian functions to the ratiometric contrast values of each standard using the DiscoverMP v2.5 software (Refeyn). In a similar fashion, DONSON proteins, were diluted ten-fold into Dulbecco PBS buffer to achieve a final concentration of 3 to 5 nM. Mass photometry videos were recorded for one minute and analysed in the DiscoverMP v2.5 software.

### Sample preparation and data collection for negative stain electron microscopy

Negative stain EM (NS-EM) samples were prepared on 300-mesh copper grids with carbon film (EM Resolution C300Cu). After glow discharging the grids for 60 s at 25 mA using GloQube Plus unit (Quorum), 4 µl of the elution from the large-scale immunoprecipitation of chromatin were applied to the grids and incubated for 1 min. Excess sample was blotted away and the procedure of applying 4 µl sample followed by blotting was repeated two more times. After third application of the sample and the final blotting, grids were stained by successive stirring in three 35 µl drops of 2% uranyl acetate for 20 s each. Excess stain was removed by blotting and the grids were left to dry for a few minutes before being stored for imaging.

All NS-EM data were acquired on a FEI Tecnai LaB6 G2 Spirit transmission electron microscope, operating at 120 kV. Micrographs were collected with a Gatan Rio 4k x 4k CMOS camera at nominal magnification of 29,000 (physical pixel size 3.1 Å /pix) and within a-0.5 to -2.0 µm defocus range.

### Negative-stain EM image processing

A subset of particles was picked using a crYOLO 1.4.0^61^ model trained for previous CMG negative stain dataset and the picks were manually curated in crYOLO. This subset was then used for training a new crYOLO model for particle picking. Subsequent processing steps were carried out using Relion-3.0.4^63^ and cryoSPARC v4.2.1.^64^ In Relion-3.0.4 the CTF for each micrograph was estimated using Gctf^65^ particles were extracted from micrographs and 2D classification was performed. *Ab initio* reconstructions and refinements were performed in cryoSPARC v4.2.1.^64^

### Sample preparation and data collection for cryo-EM

Graphene oxide dispersion was prepared by diluting 10 µl of the stock (2 mg/ml, Sigma Aldrich 763705) in 80 µl of water, resuspending the mixture by pipetting and spinning down remaining aggregates at 650 g for 1.5 min in a bench centrifuge. Clear top layer was then carefully transferred to a new tube. UltrAuFoil R1.2/1.3 grids (Quantifoil Micro Tools) were glow discharged for 5 min at 45 mA using GloQube Plus unit (Quorum), 4 µl of freshly prepared graphene oxide dispersion was applied to each grid and incubated for 2-3 min. After blotting graphene oxide, three droplets of water, 20 µl each, were sequentially picked up with the grid and blotted away, the first two with the front side of the grid (where graphene oxide had been applied) and the last one with the back side of the grid. Grids were then dried by incubating at room temperature before the sample was applied.

Grids with freshly applied graphene oxide were prepared for cryo-EM in a Vitrobot Mark IV (FEI Thermo Scientific) by triple application of the elution sample from the large-scale immunoprecipitation of chromatin and subsequent freezing in liquid ethane. 4 µl of the sample was applied on grids, incubated for 1 min at 22°C at 100% humidity, quickly blotted away (0.5 s blotting time), followed by two more rounds of sample application and blotting. After the third sample application and incubation, excess sample was blotted away for 4.5 s and grids were plunged into liquid ethane.

Cryo-EM data collection was conducted on an in-house Thermo Scientific Titan Krios transmission electron microscope operating at 300 kV, equipped with a Thermo Scientific Falcon 4i direct electron detector camera and Selectris energy filter. EPU software (Thermo Scientific) was used for automatic acquisition of movies in counting mode with physical pixel size of 0.95 Å and a total electron dose of 33 e/Å ^2^ during 5.44 s exposure time. 1674 EER frames were initially collected per movie and they were subsequently processed in 27 fractions, with 1.22 e/ Å ^2^ per fraction. A total of 82,135 movies were collected in a single session using an energy filter slit width of 10 eV and a defocus range set at -1.3 to -3.4 µm. First 7,002 movies were collected with a defocus range set at -1.3 to -2.8 µm.

### Cryo-EM image processing

RELION-4.0.0^62^ and cryoSPARC v4.2.1 were used for image processing (Figure S2). The movies were corrected for drift, dose-weighted and the contrast transfer function (CTF) parameters were estimated using CtfFind-4.1^66^ in RELION. Particles were first manually picked in cryoSPARC on a small subset of 28 micrographs across the defocus range and these 594 particles were used for creating an initial Topaz-0.2.5^67^ model for picking, through cryoSPARC. The model was then improved through several iterative cycles consisting of particle extraction (binned by two, with box size 256 px at 1.9 Å /px), cleaning up with 2D classifications in cryoSPARC or RELION and training of a new Topaz model with cleaned-up particles used as an input. To obtain better 2D-average-based separation of good particles from noise, contamination, particle aggregations or particles close to the edge of graphene oxide layers, the subset of micrographs used for iterative cycles of particle picking, extraction and 2D classifications was increased to 1,060, at first instance, and then to 28,744 micrographs. Topaz models were trained on a smaller subset of 60 or 108 micrographs with 1,000–3,000 input particles, and input particles were manually curated before training to remove obviously wrong picks. The final Topaz model (Topaz model #1) was trained on a subset of 108 micrographs across the defocus range, with 2,488 particles. Another Topaz model (Topaz model #2) was trained for picking particles in low-defocus micrographs and the training was conducted on a subset of 85 micrographs with 1822 particles and a defocus range between -1.3 and -1.9 µm. The full set of collected micrographs was curated in RELION based on CTF parameters (rlnCtfMaxResolution and rlnCtfFigureOfMerit) to remove poor-quality micrographs and micrographs without a graphene oxide coverage. This yielded a total of 67,761 micrographs that were subsequently divided in two subsets by defocus values: a subset of 56,876 micrographs with defocus ≤ -1.9 µm (high-defocus subset), and a subset of 10,885 micrographs with defocus > -1.9 µm (low-defocus subset). Using the Topaz model #1, 3,560,024 particles were picked from the high-defocus subset of micrographs. Similarly, using the Topaz model #2, 580,279 particles were picked from the low-defocus subset, making the total number of particles initially picked from the whole dataset 4,140,303. 2D-classification-based cleanup of the particles from the two subsets of micrographs was performed separately.

Using RELION, the high-defocus subset of micrographs was divided into seven groups and picked particles were extracted with a binning factor of three and a box size of 180 px (2.85 Å /px). The respective seven groups of particles were then subjected to successive rounds of 2D classifications in parallel to remove contaminants, aggregated and smaller particles as well as particles close to the edge of graphene oxide layers. The remaining particles were joined and re-extracted with a binning factor of two and a box size of 294 pix (1.9 Å /px). The low-defocus subset of micrographs was divided into two groups and the proceeding steps of particle extraction, several rounds of 2D classification and final joining and re-extraction of remaining particles were conducted in the same way as described for the high-defocus subset of micrographs. In the end, re-extracted particles coming from the high- and the low-defocus subset were joined, totalling 954,780 particles. These particles were submitted for 3D multi-reference classification with image alignment in RELION, using regularisation parameter T value of four, C1 symmetry and four low-pass filtered reference maps: (1) a double CMG-DONSON (dCMGDo) map generated by *ab initio* reconstruction and homogeneous refinement in cryoSPARC of 34,344 particles binned by two, coming from the highest-resolution dCMGDo 2D classes obtained during preliminary processing of the first 33,441 micrographs of this dataset; (2) a single CMG map generated by *ab initio* reconstruction, followed by heterogeneous and homogeneous refinements (C1 symmetry) in cryoSPARC, from a small, trial cryo-EM dataset (2301 micrographs) of the same sample collected on our in-house Thermo Scientific Talos Arctica transmission electron microscope; the remaining two reference maps, (3) and (4), which were poorly defined and did not look like CMG, were among those generated by *ab initio* reconstruction and refinements in cryoSPARC of particles binned by two obtained during preliminary processing of the first 33,441 micrographs. In this way, particles of the dCMGDo complex (219,456 particles; 23%) and particles of the single CMG (361,156 particles; 37%) were separated from the particles with poor features that did not contain complete CMG.

Particles from the dCMGDo 3D class (219,456 particles) were unbinned and re-extracted in RELION with a box size of 540 pix (0.95 Å /px), followed by *ab initio* reconstruction, heterogeneous and non-uniform refinement with applied C2 symmetry) in cryoSPARC. The refined map was low-pass filtered and used as a reference map for a 3D classification in RELION of the unbinned dCMGDo set containing 219,456 particles, performed with image alignment, C1 symmetry, regularisation parameter T value of four and with two classes. A 3D class with 128,581 particles (56%) was selected, auto-refined using C2 symmetry and post-processed in RELION to 4.2 Å resolution. These particles were then subjected to several rounds of CTF refinement and auto-refinement (C2 symmetry), as well as to two rounds of Bayesian polishing. CTF-refined and polished dCMGDo particles were then further processed in cryoSPARC, by performing *ab initio* reconstruction and heterogeneous refinement with two classes and C1 symmetry, which yielded a new set of 100,667 particles. These particles were then subjected to non-uniform refinement, with applied C2 sym-metry alignment, per-particle defocus optimisation and per-group CTF parameters optimisation, leading to a 3.2 Å resolution map. Further local refinement with a mask encompassing the whole dCMGDo complex, with applied C2 symmetry and pose/shift gaussian prior during alignment, resulted in a consensus map with improved resolution of 3.1 Å, sharpened with *B* factor of -93 Å ^2^.

The set of 128,581 CTF-refined and polished dCMGDo particles was auto-refined (C2 symmetry) and post-processed in RELION yielding a 4.07 Å resolution map. To better resolve MCM ATPase domains that featured conformational heterogeneity, a C2 symmetry expansion procedure was applied, followed by particle subtraction in RELION, where a mask encompassing ATPase domains of one MCM hexamer was used to subtract all the remaining signal. The mask was created from the auto-refined dCMGDo map, after segmentation and subtraction of all segmented regions except one MCM-ATPase in UCSF Chimera.^70^ Subtracted particles were then subjected to focused 3D classification without image alignment, using the same mask applied in particle subtraction, with ten classes, C1 symmetry and regularisation parameter T value of 16. Two 3D classes with 144,989 particles were selected and the particles were submitter to local refinement in cryoSPARC with applied C1 symmetry, pose/shift gaussian prior during alignment and dynamic masking. This yielded the MCM-ATPases map at 3.5 Å resolution, sharpened with *B* factor of -113 Å ^2^.

Particles from the single CMG subset (361,156) were re-extracted with a box size of 240 pix (1.9 Å /px) and submitted for two rounds of 3D classification in RELION, with four and two classes respectively, regularisation parameter T value of four, C1 symmetry, applied image alignment and with the low-pass filtered single CMG map from the screening Talos Arctica dataset mentioned earlier used as a reference. In between two rounds of 3D classification, the particles were unbinned (box size of 480 px, with 0.95 Å /px). 3D classes where single CMG looked isotropic were selected (137,591 particles) and the particles were submitted to cryoSPARC for *ab initio* reconstruction and non-uniform refinement with per-particle defocus optimisation, per-group CTF parameters optimisation and with C1 symmetry. Finally, local refinement with a mask encompassing the whole complex, applied pose/shift gaussian prior during alignment and with C1 symmetry was performed. This resulted in the 3.0 Å resolution map of a single CMG, sharpened with B-factor of -91 Å ^2^.

### Model building and refinement

We used AlphaFold-predicted^48^ models to initially place all *Xenopus laevis* CMG subunits into the sharpened consensus map of the dimeric CMG complex: Mcm2 (AF-P55861-F1), Mcm3 (AF-P49739-F1), Mcm4 (AF-P30664-F1), Mcm5 (AF-P55862-F1), Mcm6 (AF-Q5FWY4-F1), Mcm7 (AF-Q7ZXB1-F1), Pfs1 (AF-Q7ZT47-F1), Psf2 (AF-Q7ZT46-F1), Pfs3 (AF-Q7ZT01-F1), Cdc45 (AF-Q9YHZ6-F1) and Sld5 (AF-Q7ZT48-F1). AlphaFold Multimer was used to predict the interaction between DONSON and Mcm3 or Sld5. We first rigid-body docked protein chains comprising one CMGDo monomer into the corresponding segments of the EM density using UCSF Chimera.^70^ Since inter-domain movements within MCM subunits were apparent, they were fitted as separate rigid bodies (A domain, B/C domain, ATPase domain and winged-helix domain (WHD)). After rigid-body docking, all subunits were fitted into the density using the jiggle-fit tool and flexible fitting in real space (Geman-McClure Alpha set to 0.1) with all-chain self-restraints applied (4.3 Å). This was performed in *Coot*.^68^ For the N-terminal domains of MCM subunits, Pfs1, Psf2, Pfs3, Cdc45 and Sld5 sharp-ened consensus map was used. MCM ATPase and WHD domains were fitted to the sharpened ATPase map. Any parts of the predicted model that comprised unstructured regions or corresponded to poorly resolved regions in the map were trimmed off at this point. In particular, N-terminal hairpins (NtHp) of Mcm5, Mcm6 and Mcm7 as well as helix-2 insert (h2i) and pre-sensor 1 β-hairpin (PS1BH) elements in Mcm4 and Mcm7 could not be modeled. After flexible fitting, we used *Coot* to optimize the fit of well-resolved chain fragments to their corresponding maps (map weight set to 10), applying torsion and Ramachandran restraints to maintain back-bone and sidechain geometry. Due to the poor density of the Mcm4 ATPase and Mcm6 ZnF regions, only flexible fitting and no manual adjustments were performed for these regions. Residual density for Mcm5 WHD was visible, but it was not included in the final model due to poor map quality in that region. The resolution of the ATPase map was sufficient to include nucleotides in the ATPase sites, although the exact nucleotide state could not have been established. We therefore assigned ATP to all occupied sites (all except for unoccupied 7/4 site), but we cannot rule out the possibility that they represent ADP or mixed ATP/ADP states. Prior to structure refinement, we fitted a second CMGDo monomer (without MCM ATPase) into the symmetry-equivalent part of the consensus reconstruction to create the complete symmetry-expanded dCMGDo complex. We then refined the structures in real space with *PHENIX*^69^ using default settings and rotamer fit set to ‘outliers’. The quality of the resulting atomic models was evaluated with MolProbity^77^ and Comprehensive validation (cryo-EM) tools, both integrated into *PHENIX* (summarized in Table 1).

### Visualization of models and maps

UCSF Chimera^70^ and UCSF ChimeraX^71^ were used for visualizing all maps and models in this study. ChimeraX was also used for making figures.

## Quantification and Statistical Analysis

### DNA synthesis analysis

For quantification of replication efficiency between different experimental repeats in different extracts, the quantity of DNA replicated at the end of the reaction in IgG-depleted extract was set as 100% and the remaining values normalised to this. The mean with SEM was plotted using GraphPad Prism 9.2 or 10. Statistical significance of differences between time-courses replication curves were estimated using 2way ANOVA comparing each time to IgG-depleted replication curve. For titration of recombinant DONSON, paired, nonparametric, two-tailed t-test was performed using GraphPad Prism 10.

### EM data analysis

Quantification, statistical analysis and validation pertaining to processing of negative stain and cryo-EM images are implemented in the software described in the image processing section of the methods details. Global resolution estimation of refined cryo-EM maps are based on the 0.143 cutoffs of the Fourier Shell Correlation between two half maps refined independently.

## Supplementary Material

Video S1

Supplementary Information

## Figures and Tables

**Table 1:** *Xenla* CMG cryo-EM data collection and processing

	*Xenla* sCMG consensus (EMDB: EMD-18195)	*Xenla* dCMGDo consensus (EMDB: EMD-18191) *Xenla* dCMGDo without ATPase (PDB: 8Q6O)	*Xenla* dCMGDo MCM ATPase (EMDB: EMD-18192) *Xenla* dCMGDo MCM ATPase (PDB: 8Q6P)
Data collection and processing			
Nominal magnification	130,000	130,000	130,000
Voltage (keV)	300	300	300
Electron exposure (e^–^/Å^2^)	33	33	33
Defocus range (μm)	–1.3 to –3.4	–1.3 to –3.4	–1.3 to –3.4
Pixel size (Å)	0.95	0.95	0.95
Symmetry imposed	C1	C2	C1
Initial particle number	4,140,303	4,140,303	4,140,303
Final particle numbers	137,591	100,667	144,989
Global map resolution at 0.143 FSC threshold (Å)	3.03	3.14	3.53
Map resolution range at 0.5 FSC (Å)^a^	2.5–9.5	2.7–8.3	2.5–8.5
Map sharpening *B*-factors (Å^2^)	–91	–93	–113
Model refinement^b^			
Model-map CC (CC_mask_/CC_box_/CC_peaks_/CC_volume_)	-	0.81/0.66/0.56/0.81	0.78/0.55/0.35/0.77
Model-map FSC, 0.143 threshold (A) (masked/unmasked)	–	3.1/3.1	3.5/3.7
Model-map FSC, 0.5 threshold (Å) (masked/unmasked)	–	3.4/3.6	3.9/6.4
Model composition			
Non-hydrogen atoms	–	51,983	16,795
Protein residues	–	6,454	2,104
Ligands	–	10 × Zn^2+^	5 × ATP
RMS deviations			
Bond lengths (Å)	–	0.002	0.002
Bond angles (°)	–	0.492	0.484
Mean *B*-factors (Å^2^)			
Protein	–	122.07	108.87
Ligands	–	260.17	83.98
Validation^c^			
MolProbity score	–	1.35	1.35
Clashscore	–	4.9	5.07
Poor rotamers (%)	–	0.05	0.05
CaBLAM outliers (%)	–	1.20	1.48
Ramachandran plot^c^			
Favored (%)	–	97.6	97.6
Allowed (%)	–	2.4	2.4
Outliers (%)	–	0.0	0.0

a Estimated with cryoSPARC.^64^

b Calculated with comprehensive validation (cryo-EM) tool in *PHENIX*.^69^

c MolProbity^77^ validation in *PHENIX*.

## Highlights

- Purified complex from replicating chromatin frog egg extracts is cryo-EM tractable
- Structure of a double CMG bound by a DONSON dimer
- MCM double hexamer misaligned and ready to replicate bidirectionally
- Patients’ mutations impairing DONSON dimerization affect DNA replication

## In brief

Cvetkovic, Passaretti, et al. determine the cryo-EM structure of a double CMG bound by dimeric DONSON, explaining how the CMG helicase is assembled symmetrically in metazoans. MCM rings become misaligned and poised to cross paths supporting replication origin activation. Disrupting DONSON dimer affects DNA replication, explaining etiology of primordial dwarfism.
