# Supplementary material for: The structural mechanism of dimeric DONSON in replicative helicase activation
Source: Mol Cell. Author manuscript; Available in PMC 2024 Nov 11. (PMC7616792; doi:10.1016/j.molcel.2023.09.029)
Supplement: Supplementary Information [file EMS199717-supplement-Supplementary_Information.pdf]

Molecular Cell, Volume *83*

Supplemental information

The structural mechanism of dimeric DONSON in replicative helicase activation

Milos A. Cvetkovic, Paolo Passaretti, Agata Butryn, Alicja Reynolds-Winczura, Georgia Kingsley, Aggeliki Skagia, Cyntia Fernandez-Cuesta, Divyasree Poovathumkadavil, Roger George, Anoop S. Chauhan, Satpal S. Jhujh, Grant S. Stewart, Agnieszka Gambus, and Alessandro Costa

Supplementary information

The structural mechanism of dimeric DONSON in replicative helicase activation

Milos A. Cvetkovic, Paolo Passaretti, Agata Butryn, Alicja Reynolds-Winczura, Georgia Kingsley, Aggeliki Skagia, Cyntia Fernandez-Cuesta, Divyasree Poovathumkadavil, Roger George, Anoop S. Chauhan, Satpal S. Jhujh, Grant S. Stewart, Agnieszka Gambus, Alessandro Costa

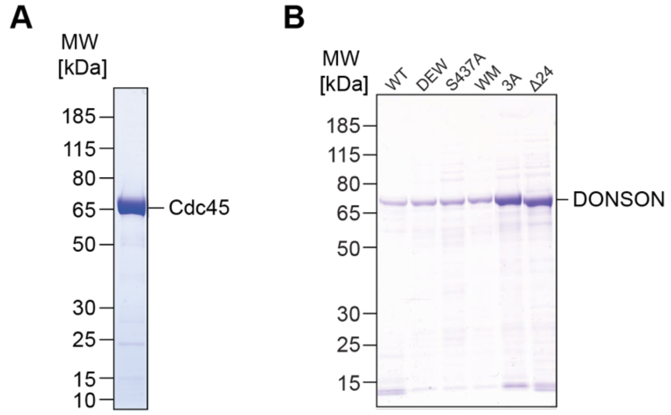

Figure S1. Recombinant proteins created in this study, related to Figure 1. Purified proteins resolved on SDS-PAGE gel and stained with Coomassie: **(A)** *Xenla* Cdc45-TEV-His_{10}-$FLAG_5$. **(B)** *Xenla* DONSON variants: wild-type (WT); Asp374Ala, Glu377Ala and Trp381Ala (DEW); Ser437Ala; Trp234Leu, Met463Thr (WM); Val473Ala, Arg476Ala, Tyr481Ala (3A); deletion of residues 1 to 24 (Δ24).

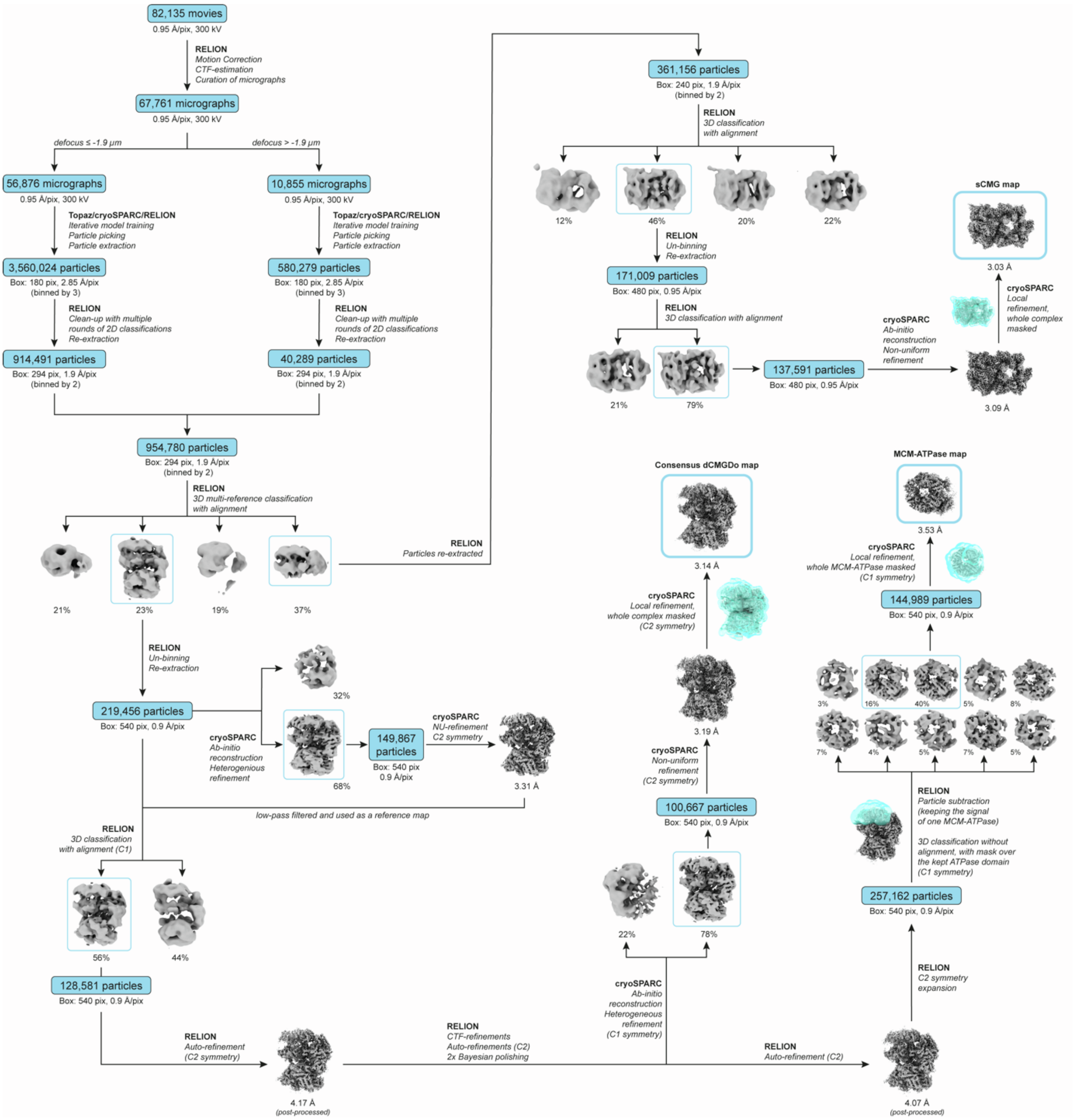

Supplementary Figure 2. Cryo-EM image processing pipeline, related to Figure 2.

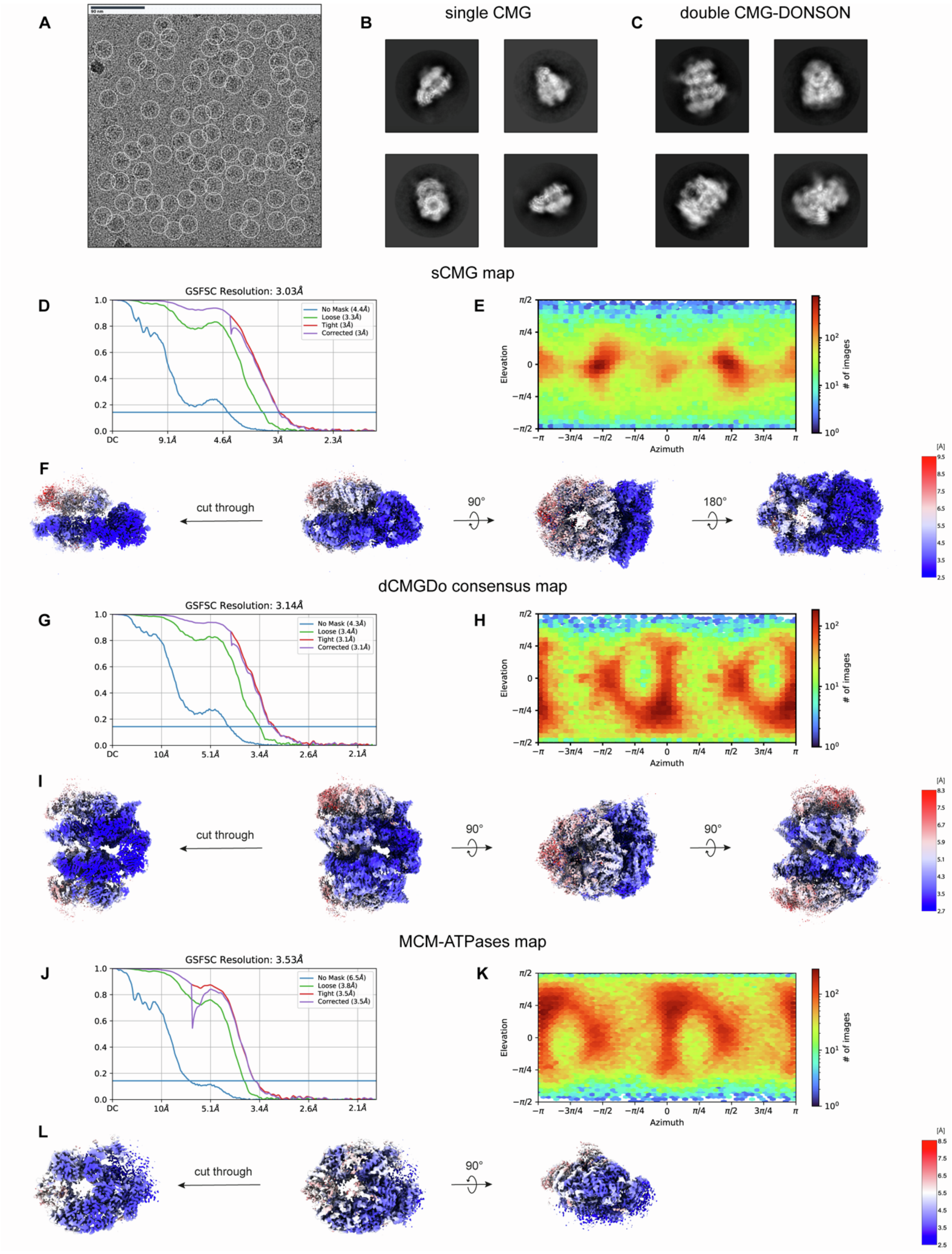

Supplementary Figure 3. Cryo-EM analysis of single CMG and dCMGDo, related to Figure 2. **(A)** A representative micrograph. **(B)** 2D averages of single CMG. **(C)** 2D averages of dCMGDo. **(D)** Fourier shell correlation plot for single CMG. **(E)** Angular distribution for single CMG. **(f)** Cryo-EM map coloured according to local resolution for single CMG. **(G)** Fourier shell correlation plot for dCMGDo consensus map. **(H)** Angular distribution for dCMGDo consensus map. **(I)** Cryo-EM map coloured according to local resolution for

dCMGDo consensus map. **(J)** Fourier shell correlation plot for MCM ATPase map isolated from the dCMGDo. **(K)** Angular distribution for MCM ATPase map. **(L)** Cryo-EM map coloured according to local resolution for MCM ATPase map.

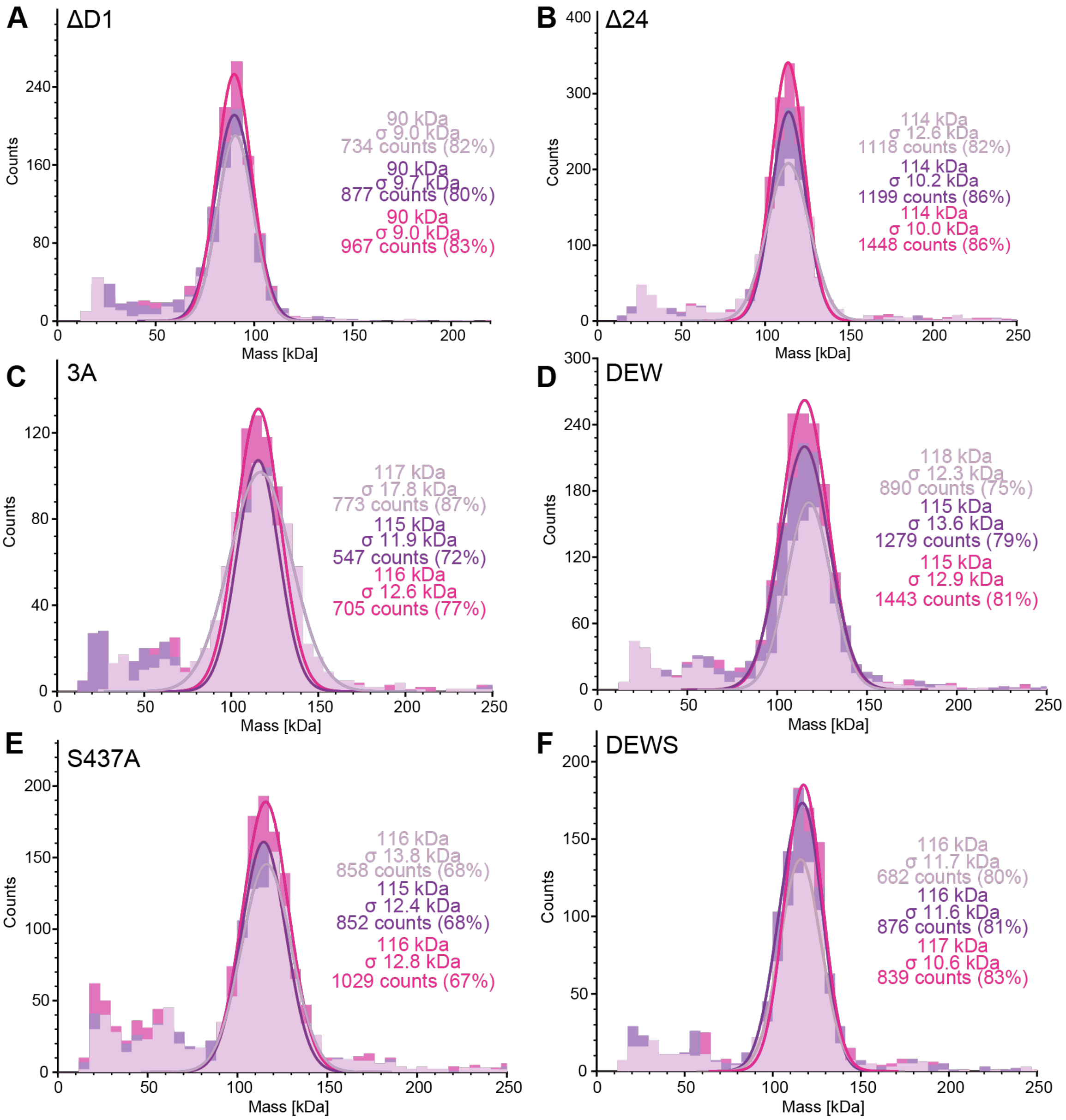

Supplementary Figure 4. Mass photometry analysis of various *Xenopus laevis* DONSON mutants, related to Figures 3 and 4. All variants shown in this figure maintain a dimeric character despite the amino acid changes. **(A)** Truncation of residues 1 to 155 (D1). **(B)** Truncation of residues 1 to 24 (Δ24). **(C)** Val473Ala, Arg476Ala, Tyr481Ala (3A). **(D)** Asp374Ala, Glu377Ala, Trp381Ala (DEW). **(E)** Ser437Ala. **(F)** Asp374Ala, Glu377Ala, Trp381Ala, Ser437Ala (DEWS).

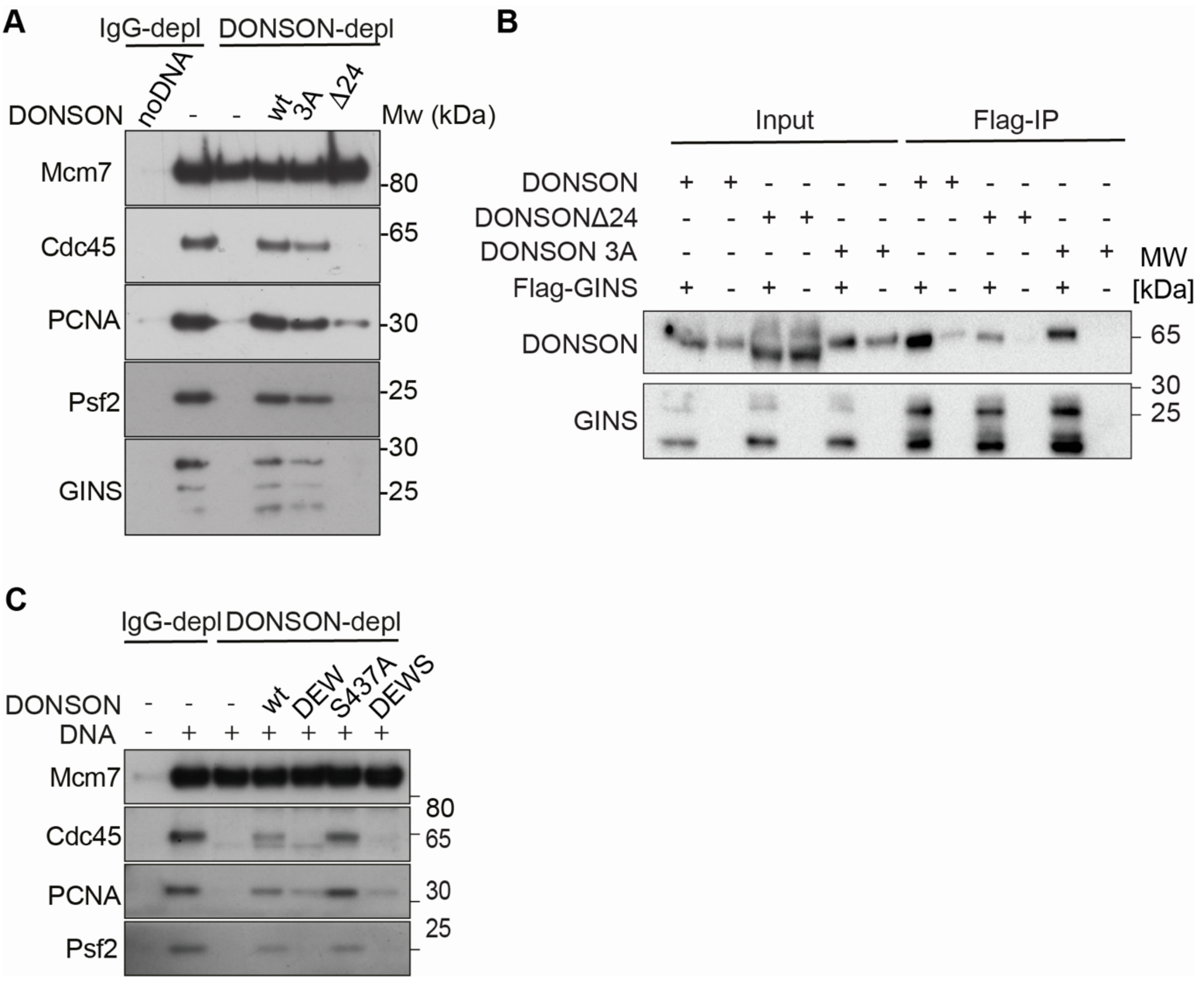

Figure S5. Functional analysis of DONSON mutants, related to Figures 3 and 4. (A) DNA replication reaction was set up in IgG- or DONSON-depleted extract optionally supplemented with 100 nM recombinant wild type or indicated DONSON mutants. Chromatin was isolated at 60 min of replication reaction. Chromatin bound factors were resolved on SDS-PAGE and immunoblotted with indicated antibodies. "No DNA" control served as chromatin specificity control. **(B)** Recombinant purified DONSON WT, Δ24 or 3A mutants were mixed and incubated with *Xenopus laevis* GINS complex expressed in *E. coli* and purified via His_6-FLAG-tagged Sld5. M2-FLAG beads were used to immunprecipitate GINS. Co-immunoprecipitating proteins were analysed by immunoblotting with indicated antibodies. **(C)** DNA replication reaction was set up in IgG- or DONSON-depleted extract optionally supplemented with 100 nM recombinant wild type or indicated DONSON mutants. Chromatin was isolated at 60 min of replication reaction. Chromatin bound factors were resolved on SDS-PAGE and immunoblotted with indicated antibodies. "No DNA" control served as chromatin specificity control.

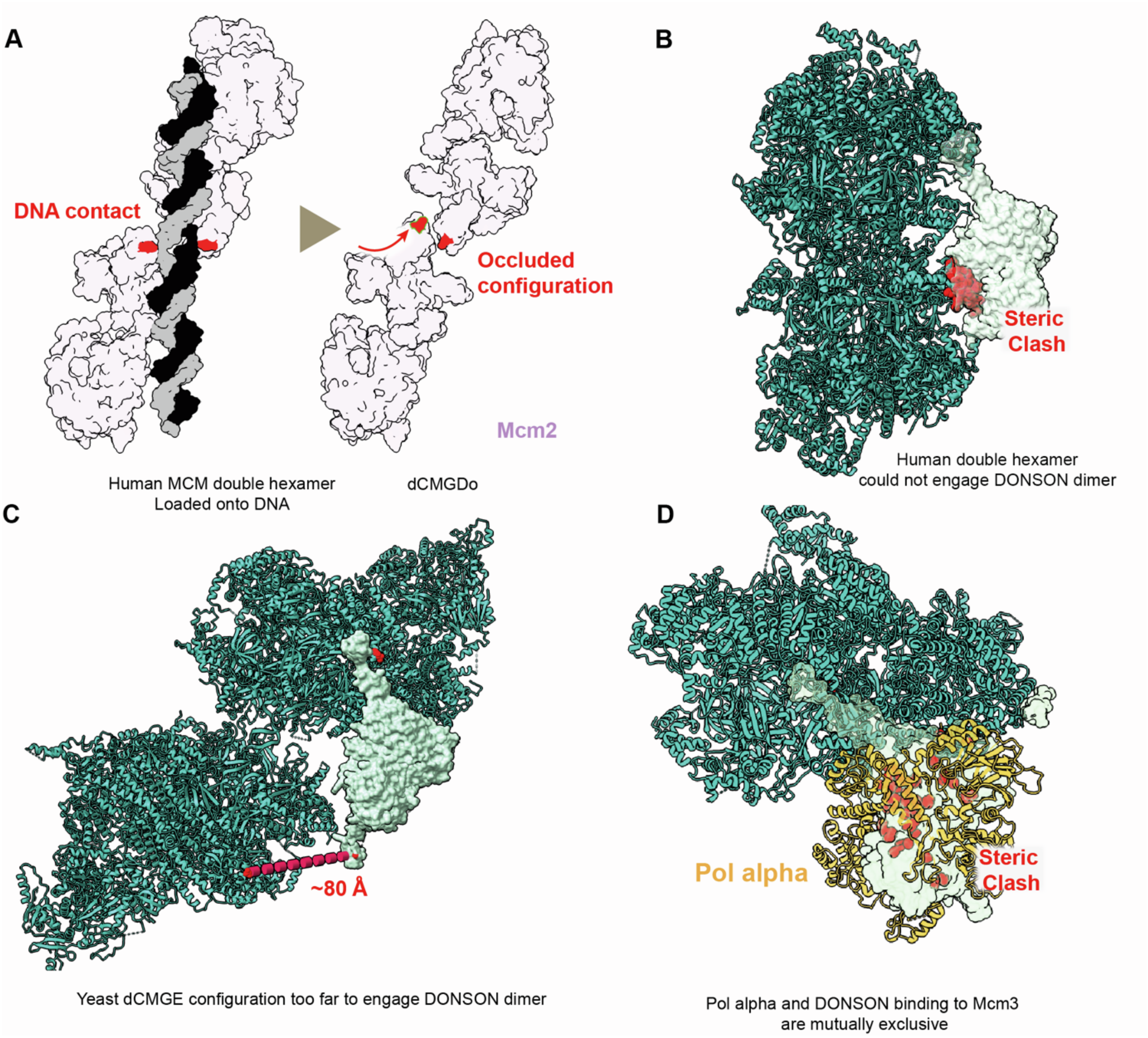

Supplementary Figure 6. Comparison between the dCMGDo structure and previously published structures, related to Figure 5. CMG is colored in teal. DONSON is light green. **(A)** dCMGDo shuts the duplex DNA harbouring MCM channel at the N-terminal dimerisation interface. The side of the Mcm2 ZnF domain that engages duplex DNA in the human double hexamer (PDB entry TW1Y) is backed against the cognate Mcm2 ZnF element in dCMGDo. **(B)** Mcm3 engagement by DONSON, as observed in dCMGDo, is incompatible with double hexamer formation as observed with the DNA loaded human (PDB entry TW1Y), or yeast, MCM. **(C)** The double CMG configuration observed in the yeast dCMGE (7Z13) is too dilated to allow concomitant Mcm3 engagement of both protomers in the DONSON dimer. **(D)** Pol alpha (PDB entry 8B9D) and DONSON compete for the same binding site on Mcm3.

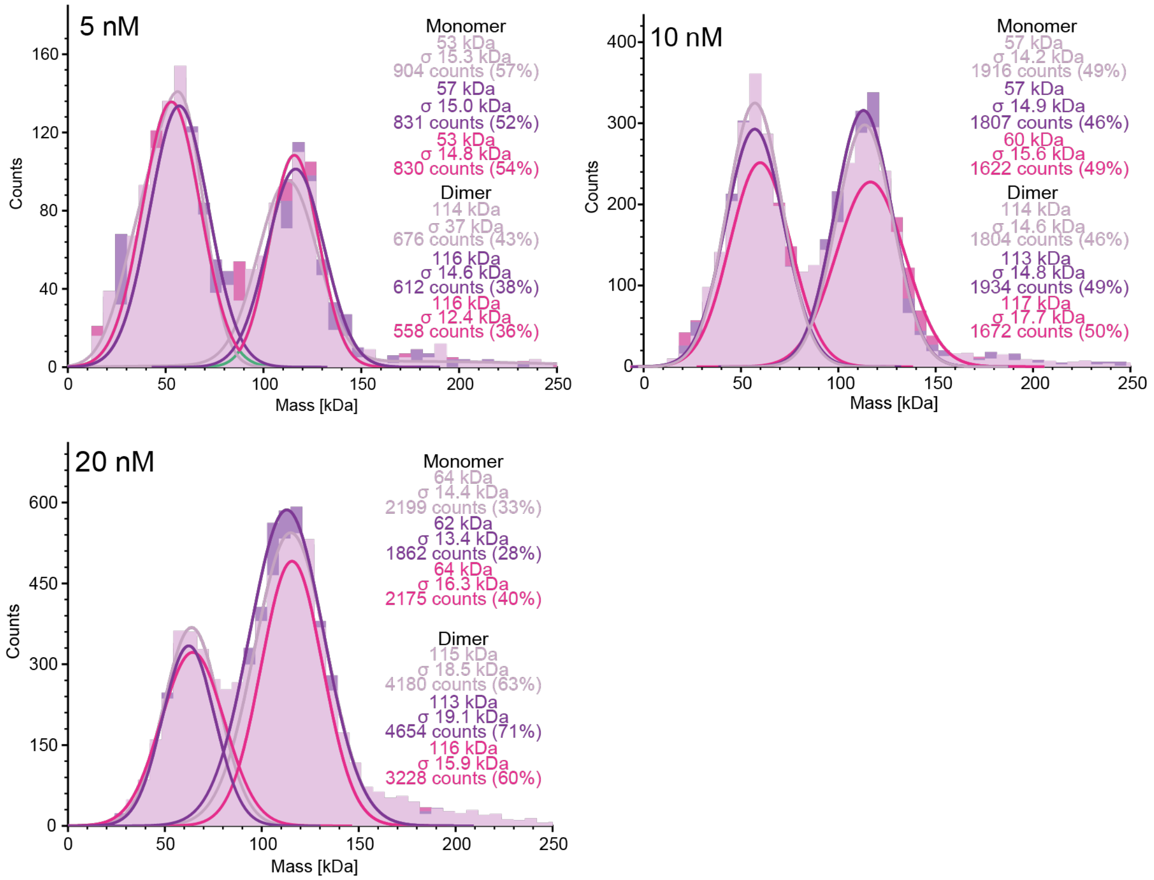

Supplementary Figure 7. Mass photometry analysis of DONSON Trp234Leu, Met463Thr (WM) mutant, related to Figure 6. *Xenopus laevis* DONSON amino acid changes homologous to patients' muations in humans destabilise the homodimerization interface. A dilution experiment highlights the tendency of the WM mutant to monomerise as concentrations drop from 20 to 5 nM.

	Protein	Molecular Weight (kDa)	Spectral Counting (SpC)
**CMG**	Mcm2	100	134
	Mcm3	90	127
	Mcm4	97	78
	Mcm5	82	101
	Mcm6	93	138
	Mcm7	82	72
	Cdc45	66	181
	Psf1	23	11
	Psf3	24	8
	Psf2	21	2
	Sld5	26	4
**Replisome**	Polα (POLA1)	165	29
	Polδ (POLD1)	125	41
	Polδ (POLD2)	49*	3*
	Polδ (POLD3)	50	11
	Polε (POLE1)	265	107
	Polε (POLE2)	60	10
	Rts (RecQ4)	169	15
	AND-1	125	69
	PCNA	29	9
	Claspin	146	8
	Timeless	149	18
	Spt16	118	37
	SSRP1	79	18
**Initiation factors**	Mcm10	95	28
	MTBP	96	4
	Orc1	100	4
	Orc3	81	2
	Orc5	52	4
	Rif1	257	70
	Treslin	221	33
	TopBP1	169	6
	DONSON	64	5

Supplementary Table 1. Mass spectrometry analysis of a Cdc45-containing replication intermediate, related to Figure 1. Proteins co-immunoprecipitating with Cdc45-TEV-His_{10}-$FLAG_5$ isolated from chromatin replicating in *Xenopus laevis* egg extract supplemented with aphidicolin and caffeine (as per Figure 1D) were analysed by mass spectrometry. Selected

replication factors are presented with molecular weight and total spectral count. Full data available on PRIDE. (*Xenopus tropicalis proteins).
